# Estimating sectoral livestock biomass and stock value using data from national diseases eradication programs: a case study based on the Irish cattle herd from 2011 to 2021

**DOI:** 10.3389/fvets.2025.1648948

**Published:** 2025-10-02

**Authors:** Emma-Jane Murray, Eoin Ryan, Jonathan Rushton, Jamie A. Tratalos, Jonas Brock, Elaine Tarrant, Sharon Walshe, David A. Graham, Damien Barrett, Timothy Geraghty, Conor McAloon

**Affiliations:** ^1^UCD School of Veterinary Medicine, University College Dublin, Dublin, Ireland; ^2^Global Burden of Animal Diseases, The Roslin Institute University of Edinburgh, Midlothian, United Kingdom; ^3^Animal Health and Welfare Division, Department of Agriculture, Food and the Marine, Dublin, Ireland; ^4^Centre for Veterinary Epidemiology and Risk Analysis, UCD School of Veterinary Medicine, University College Dublin, Dublin, Ireland; ^5^Animal Health Ireland, Carrick on Shannon, Ireland; ^6^ERAD Division, Department of Agriculture, Food and the Marine, Celbridge, Co. Kildare, Ireland; ^7^Glenythan Vet Group, Aberdeenshire, United Kingdom; ^8^Dunnydeer Veterinary Group, Aberdeenshire, United Kingdom

**Keywords:** cattle, biomass, animal health, economics, livestock

## Abstract

**Introduction:**

Livestock biomass is a denominator for a wide range of important production metrics, including productivity, environmental impact, greenhouse gas emissions, and antimicrobial usage. Accurate biomass estimates allow cross-sectoral and international comparisons for these important indices across a range of high-priority areas, which can then inform policy risk assessments and decision-making. Similarly, accurate estimates of the value of livestock are needed to monitor economic efficiency and productivity and understand the costs associated with animal health policy decisions. Previous methods to estimate biomass have relied on assigning an average liveweight for a given species and multiplying this by the number of individual animals of that species in a region. However, without taking into account the population’s demographics and structure, these approaches cannot be relied upon to accurately represent the cattle population.

**Methods:**

Using data from the Irish cattle herd as a case study, this study developed liveweight and value models and applied these models to a cattle registration and movement database to estimate the biomass (kg) and economic stock value (€) of each animal and herd, aggregated by herd type based on a herd classification tree model, and explored trends in biomass and stock value over time.

**Results:**

The Irish cattle sector biomass increased from 2,924,800 tonnes in 2011 to 3,317,100 tonnes in 2021, and the cattle sector stock value increased from €6,323.7 m in 2011 to €8,792.3 m in 2021. Furthermore, this study demonstrated the biomass and stock value within-year and between years.

**Discussion:**

We illustrate a novel approach using real-time movement data for dynamic estimates of biomass and stock value at animal-, herd- and national-level that can be applied in countries with existing animal registration and movement tracing systems.

## 1 Introduction

Livestock biomass is a denominator for a wide range of important production metrics, including productivity, environmental impact, greenhouse gas emissions, and antimicrobial usage (1, 2). Accurate biomass estimates allow cross-sectoral and international comparisons for these important indices across a range of high-priority areas, which can then inform policy risk assessments and decision-making.

Previous methods to estimate biomass have relied on assigning an average liveweight for a given species and multiplying this by the number of animals of that species in a region. For example, the population correction unit (PCU), introduced by the European Medicines Agency (EMA), is used to calculate the population’s livestock biomass in relation to antimicrobial consumption. In calculating antimicrobial use metrics, the method initially involved assigning a liveweight of 450 kg per adult cow, which was then multiplied across all animals in a herd, region and country (3, 4). More recently, this approach has been refined to use additional average weights for age, and sex categories, providing a closer estimate to reality, and higher than the previous PCU (5).

Similarly, Livestock Units (LSUs, a.k.a. LUs) and Tropical Livestock Units (TLUs), as reference units for aggregating livestock from various species and ages, are established based on each animal category’s nutritional or feed/energy requirement. One LSU equates to one grazing adult dairy cow producing 3,000 kg of milk a year in the European Union (6), which is the equivalent of 650 kg in liveweight. In tropical regions, a TLU equates to 250 kg of liveweight with no specification of animal performance or activity (7).

However, variability in the livestock population and production systems within- and between countries might not be accurately represented in this average unit-based approach since they do not take into account the population’s demographics and structure (3, 8). For example, the average cattle size and biomass are dependent on the average weight of cattle of a given age and the age mix of the herd, and these factors are expected to vary within and between countries. Although the LSU allows for direct comparison between countries, differences in the within-herd and within-country animal demographics may mean that average conversion methods over- or underestimate important metrics and inaccurately capture animal protein production in agricultural systems (2).

Similarly, accurate estimates of livestock value are needed to monitor economic efficiency and productivity and to understand the costs associated with animal health policy decisions (9). Such values represent a starting point for calculating aggregated animal health losses and, following this, the economic burden of specific cattle diseases nationally (9–12). Changes in value over time may be driven by structural economic changes, conflict, and economic development, varying between countries. Knowing this change in production system, country, region, and over time provides a narrative to future agricultural production, market development, investment in animal health and welfare, environmental impact, and trade-offs (13). Notably, Schrobback, Dennis (13) highlighted the need for the total economic value to be considered when studying the value in terms of use and non-use or monetary and non-monetary. However, this study strictly focused on monetary aspects, market value and stock value.

In countries such as Ireland, where livestock are registered in identification and movement tracing systems, information on the sex, breed and age of each animal is often available and could be used to estimate biomass and stock values at animal-, herd- and/or national-level at a given point in time. These estimates could be used in comparing economic input and output, GHG emissions, antimicrobial usage, and biomass distribution and use across different cattle systems to identify the most efficient and sustainable farms and sectors (14, 15).

Using data from the Irish cattle herd as a case study, the aims of this study were to: (1) develop liveweight and value models to estimate animal mass and value, (2) apply these models to a cattle registration and movement database to estimate biomass and economic stock value over time, and (3) explore trends in these metrics over time and according to herd categories.

## 2 Materials and methods

### 2.1 Overall approach (including data sources)

**Figure 1:**
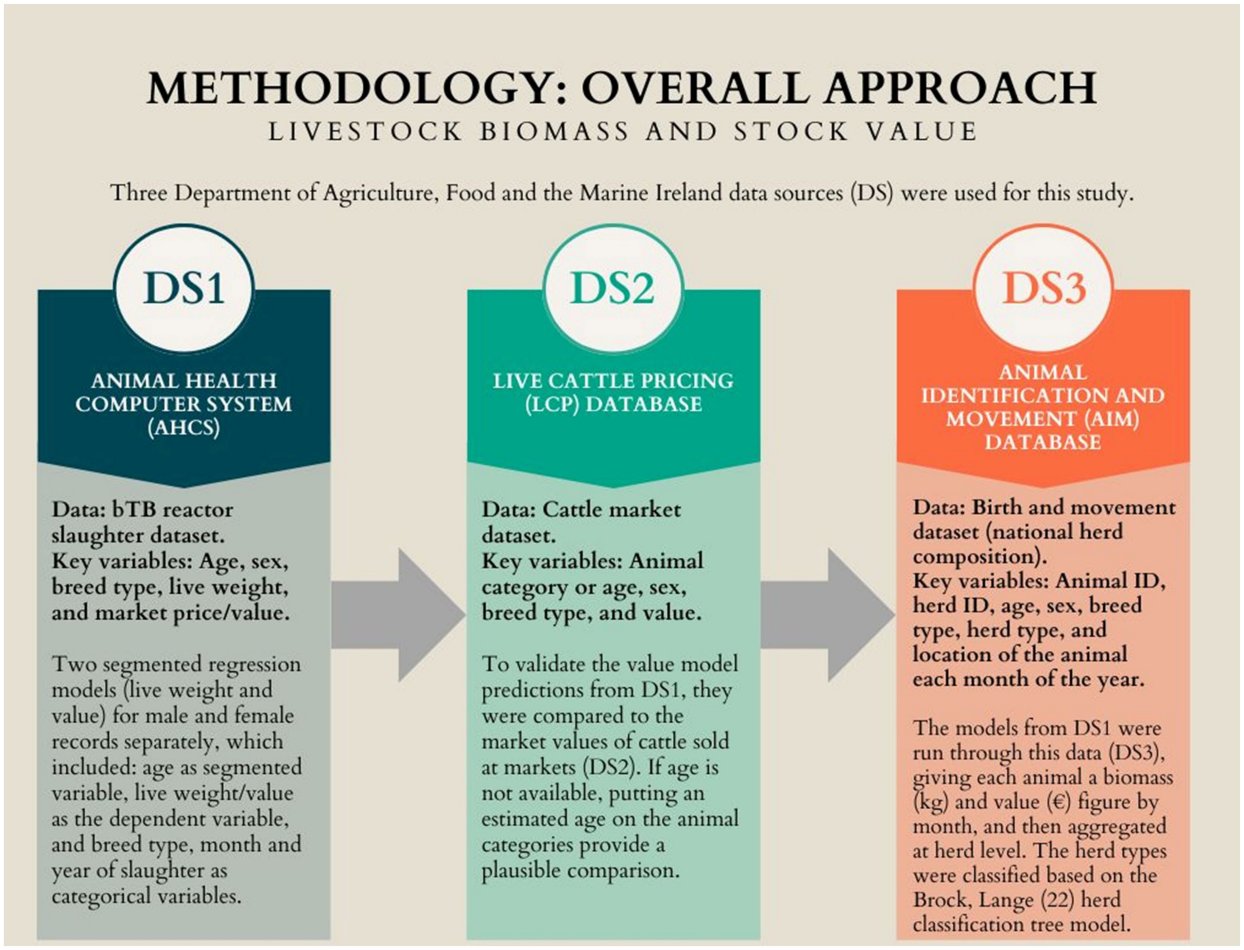
A schematic to estimate livestock biomass and stock value of animals using national databases.

This study was conducted in three phases, each utilizing separate data sources (DS) obtained from the Department of Agriculture, Food and the Marine Ireland (DAFM). Figure 1 shows a schematic of this study’s overall approach and key variables needed.

In the first phase, two models were created at the animal level to predict animal liveweight (hereafter ‘the liveweight model’) and value (‘the value model’) according to three animal characteristics: age, sex, and breed category. To do this, DS1, the Animal Health Computer System (AHCS), was used to develop models for liveweight and value. The AHCS is a computer system DAFM uses to record animal-level data relating to the bovine tuberculosis (bTB) eradication program. It records the bTB reactor slaughter data collected as part of the programs ‘On-Farm Market Valuation Scheme’ (OFMV). The OFMV is the principal compensation measure available to herdowners whose herds are affected by disease. The amount of compensation is determined through assessment by an independent Valuer or an Arbitrator (16). The data used from DS1 were animal ID, breed, animal category (i.e., calf, heifer, steer, bull, cow, pregnant heifer), sex, date of birth, date of death, eligible price, and liveweight.

The second phase consisted of validating the value model using livestock market data from the Live Cattle Pricing (LCP) database (DS2), i.e., price, breed, sex, and animal category (i.e., calf, weaned, maiden heifer, in-calf heifer, young bull feeder, light store, forward store, lactation 1–4+, finished, and cull cow). DAFM staff collect these data regularly from markets across the country at the point of auction weekly to establish parameters for compensation for bTB reactor cattle. These data are then recorded centrally on the LCP database. The outputs from the value model (developed from DS1) were compared with the livestock market valuation from DS2 to determine how well these outputs matched real-world data. This step was not conducted for the liveweight model since no suitable comparator datasets existed.

The study’s third phase applied these liveweight and value models to the demographics of the cattle population calculated from animal-level information in the AIM database (DS3) to provide liveweight and value estimates for all animals in the country and aggregate these by herd and herd type (see section 2.5 for more information on herd classification). The Animal Identification and Movement (AIM) database captures all cattle’s breed and sex, dates of birth and death, and any movements they have made to new herds (17).

Prior to data cleaning, the data were extracted for the periods 1^st^ of January 2011 to the 31^st^ of December 2021; data from DS1 included 218,669 records, DS2 524,104 records, and DS3 1,282,372,861 records.

### 2.2 Data cleaning

Animal records used for the liveweight and value models (DS1) were cleaned of identifiable data (i.e., specific animal tag numbers, herd numbers, and location). Animal records with a liveweight from 30 kg to 1,400 kg for calves, heifers, or steers and from 200 kg to 1,400 kg for bulls, cows, and pregnant heifers were retained as weights outside of those ranges were considered unrealistic and represented data entry errors. Any records missing value data (or value equals zero), liveweight, date of birth, date of death, sex, or breed were also excluded.

The breed of each animal was categorized into dairy, continental beef, and British-Irish beef categories, using the breed of the sire in the case of mixed breeds, e.g., FRX, meaning Friesian Cross was changed to FR (Supplementary Table S1). The breed category was determined using a breed list managed by DAFM (18).

### 2.3 Liveweight and value models (DS1)

The liveweight and value models used data from the cleaned bTB reactor slaughter dataset (DS1). To enable the prediction of animal liveweight, a segmented (piecewise) regression model was developed with animal age as the segmented variable, liveweight as the dependent variable and breed category, month and year of slaughter as categorical variables (19, 20). Segmented regression partitions the segmented independent variable (in this case, age) into a series of intervals determined by break-points. The relationship between this variable and the dependent variable (in this case, liveweight or value) is estimated separately for each interval.

Separate models were fitted for male and female animals given the likely different break-point positions and trajectories by sex. The number of break-points was chosen by incrementing from 1 to 3 and selecting the minimum number of break-points, beyond which only minor improvements in model fit, as assessed by the resulting R-squared value, occurred (19, 21). The value model was developed in the same way as the liveweight model, but with value as the dependent variable. Models were developed on a random sample (liveweight *n* = 600; value *n* = 1,000) of the overall dataset (liveweight *N* = 82,155; value *N* = 213,176). Sampling from the dataset was stratified by animal class (*n* = 6), with equal numbers sampled per animal class. The sample size was determined by the least frequent animal class (breeding bulls). Therefore, the sample size for animal weight was 3,600 (i.e., *n* = 600 per animal class; the liveweight model) and *n* = 6,000 (i.e., *n* = 1,000 per animal class) for animal value (the value model). The sample size was smaller for the liveweight model because there were more missing values for weight than for animal value.

### 2.4 Validation of the value model (DS2)

The value model was validated using a second separate dataset (DS2). The date of birth was not routinely recorded in this dataset. To facilitate the validation of the model, the animal categories used in DS2 were converted into an age range. Supplementary Table S2 shows the animal categories and their age ranges used in the validation. Next, the value model (DS1) was used to predict the value for each month of age in the specified age range for the median month and median year in the dataset. The average predicted value per animal category was calculated and compared with the average value in DS2.

### 2.5 Prediction and aggregation

Based on the animal characteristics, the liveweight and value models were calculated to predict the liveweight and monetary value of each animal present in the AIM database (DS3) for the first day of each month from 2011 to 2021.

For each month, predictions were aggregated by herd, summing biomass and stock value of all animals in each herd in that month. Each herd was classified using a modified decision tree based on the Brock, Lange (22) herd classification model. Herds without data for January, May and September were classified as trading, fattener or unknown (Supplementary Figure S1). Beef herds were further classified into subtypes as follows: beef pedigree (BP), beef suckling to beef (BSB), beef suckling to weanling (BSW), beef suckling to youngstock (BSY), beef suckling to youngstock non-rearing (BSY_nR). Dairy herd’s subtypes were standard dairy (D), non-rearing dairy contract rearing (DnR_C), non-rearing dairy no contract rearing (DnR_nC), and dairy rearing male calves (DRm). Store/rearing herd’s subtypes were rearing dairy females (Rdf), store beef females (Sbf), store beef males (Sbm), store beef mixed (Sbmx), and store dairy males (Sdm).

Within-year predictions were defined as the monthly average by herd type. Annual predictions were described as the annual average by herd type, and the annual average for each herd and by herd type were combined to get the overall sectoral predictions.

The value predictions, derived from the market values in DS1, were adjusted for inflation using the Consumer Price Index from the Central Statistics Office Ireland’s annual and monthly data from 2011 to 2021 (Base December 2011 = 100) (23, 24).

All data manipulation and statistical analyses were conducted in R version 4.2.2 (R Core Team, 2022) using the “tidyverse” (25) and “segmented” (19) packages.

### 2.6 Biomass comparison

Data on biomass related to the PCU and LSU were accessed from (26) and (27), respectively. The comparison analysis was conducted in Excel (28).

## 3 Results

### 3.1 Descriptive statistics—animal level

The study population consisted of 29,927,470 animals (890,809,644 records; Table 1). From 2011 to 2021, the average total number of animals in the national herd per year was 6,793,649, with an average of 6,186,611 animals in 2011 and 7,002,269 animals in 2021 throughout the year.

**Table 1:** Number of records per data source post data cleaning.

Data source	Date	Records
DS1	3^rd^ of January 2011 to the 31^st^ of December 2021	77,056
DS2	6^th^ of January 2011 to the 21^st^ of November	498,407
DS3	1^st^ of January 2011 to the 1^st^ of December 2021	890,161,857

### 3.2 Descriptive statistics—herd level

Overall, DS3 contained data on 129,939 unique herds between 2011 and 2021, of which there were an average annual distribution, with yearly variation, of 55,856 (50.0%) herds classified as beef, 16,512 (14.8%) as fattening, 15,995 (14.4%) as store/rearing, 13,101 (11.7%) as dairy, 5,238 (4.7%) as mixed production, 4,199 (3.8%) as unclassified, and 961 (0.9%) as trading. Some herds became dormant while other herds were newly established, and some herds changed herd type during the study period. Information for all herd types and subtypes are given in Supplementary Table S3.

The average annual median herd size from 2011 to 2021 was 31 for beef herds, 31 for fattening herds, 17 for store/rearing herds, 139 for dairy herds, 110 for mixed production herds, 7 for unclassified herds, and 23 for trading herds. From 2011 to 2021, the median herd size increased for all herd types, more so for dairy and mixed production herds (Supplementary Table S4).

### 3.3 Model results

The estimated coefficients from the liveweight and value models for female and male animals are shown in Supplementary Tables S5–S8. Two break-points were used for all four models and the break positions were estimated at 608 and 1878 days; 713 and 1884 days; 682 and 1,254 days; and 335 and 1,041 days for the female liveweight, male liveweight; female value and male value models, respectively.

**Figure 2:**
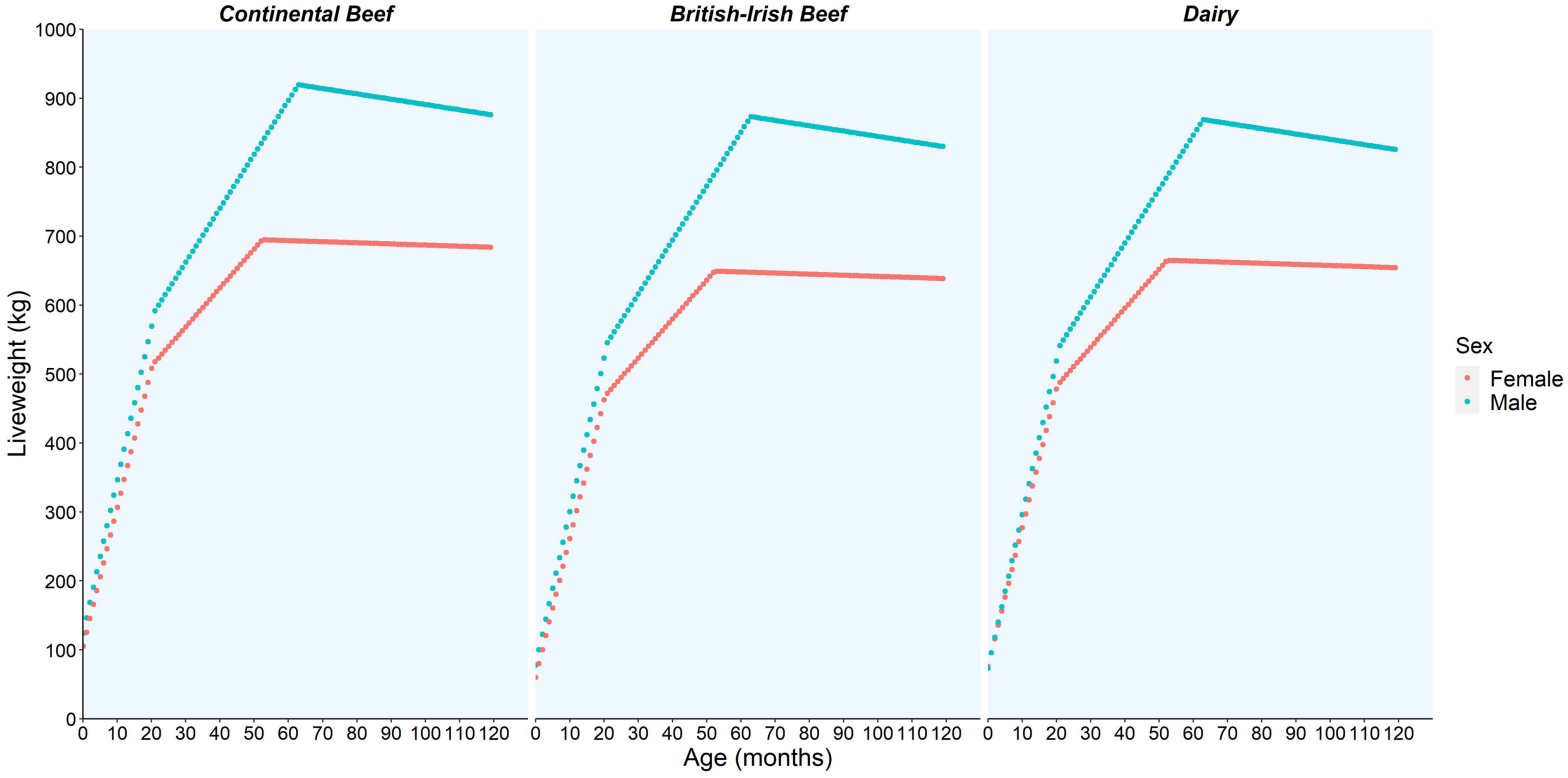
The liveweight (kg) model prediction per month of life and by sex and breed category (continental beef, British-Irish beef, and dairy) based on animals slaughtered in June of 2016 using the bTB reactor data.

**Figure 3:**
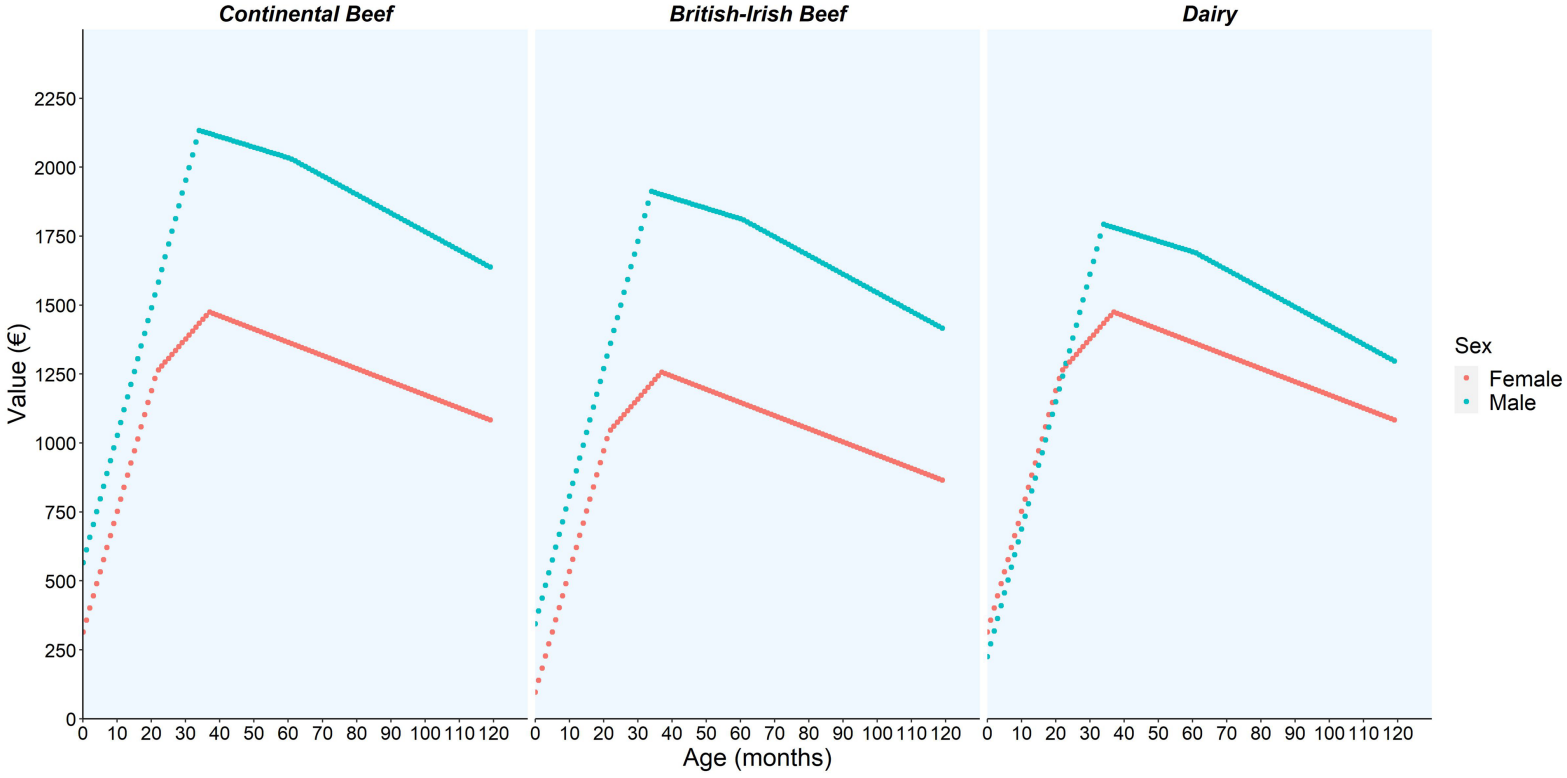
The value (€) model prediction per month of life and by sex and breed category (continental beef, British-Irish beef, and dairy) based on animals slaughtered in June of 2016 using the bTB reactor data.

Figures 2, 3 show the illustrative predictions of liveweight and value per month of life and by sex and breed based on animals slaughtered in June 2016.

### 3.4 Model validation

Table 2 shows the result of the validation against market data (DS2) for the value model (DS1) applied to dairy and beef animals. For most animal classes, predictions were generally within 10% of the market value for both dairy and beef. Overall, there appeared to be a tendency to underpredict the value of youngstock and overpredict the value of older animals, except for overpredicting beef calves, and underpredicting lactation 4 + and finished beef.

**Table 2:** Validation results of dairy and beef animals comparing the livestock market data (market value), DS2, with the value model estimates (predicted), DS1, in Euros (€).

Animal class	Market value (mean)	Predicted
Dairy		
Heifer Calf	578.20	460.40
Weaned Heifer	877.70	774.00
Maiden Heifer	950.70	1,110.00
In-calf Heifer	1,373.00	1,309.90
Lactation 1	1,606.00	1,426.00
Lactation 2	1,572.00	1,487.50
Lactation 3	1,478.60	1,539.90
Lactation 4+	1,243.70	1,320.10
Beef		
Calf	209.50	407.50
Weaned	756.40	677.10
In-calf Heifer	1,702.00	1,222.70
Young Bull Feeder	958.20	873.20
Light Store	871.50	946.60
Forward Store	1,110.40	1,301.80
Parity 1	1,342.80	1,397.30
Parity 2	1,357.10	1,449.70
Parity 3	1,369.30	1,397.20
Parity 4+	1,241.70	1,187.00
Finished	1,304.60	1,263.90
Cull Cow	1,002.10	1,283.40

### 3.5 Biomass and stock value by sector

**Figure 4:**
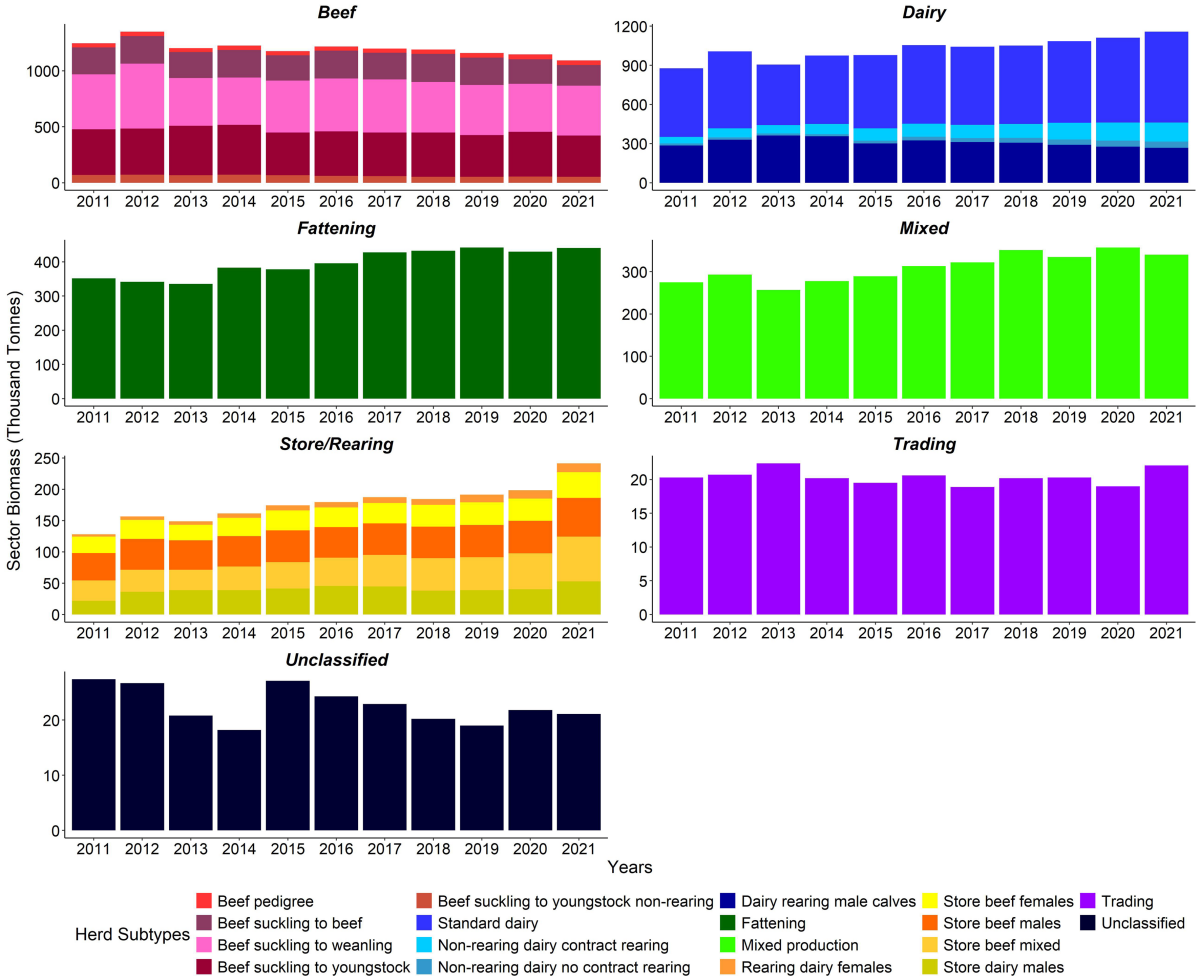
Sector biomass (‘000 tonnes) by herd type 2011–2021 (note differences in biomass scales between sectors).

Overall, the cattle sector biomass increased from 2,924,800 tonnes in 2011 to 3,317,100 tonnes in 2021 (Supplementary Table S9). Biomass for the dairy, fattening, mixed production, and store/rearing sectors followed generally upward trends (Figure 4), whereas biomass in the beef sector followed a generally downward trend from 2012 (Figure 4; Supplementary Table S9).

**Figure 5:**
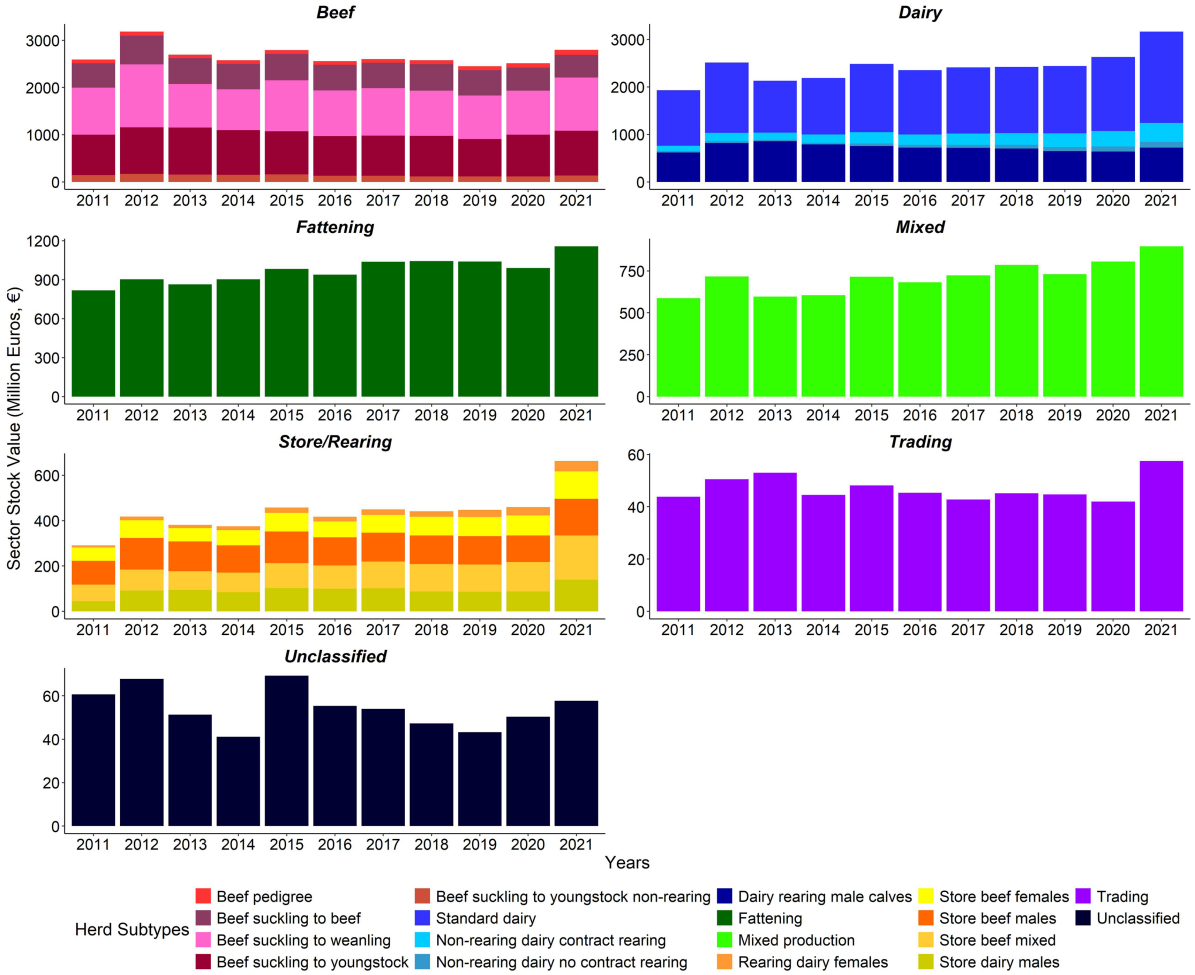
Sector stock value (€ million) by herd type 2011–2021 (note differences in value scales between sectors). Adjusted for inflation (Base December 2011 = 100) (23).

The cattle sector stock value at real prices increased from €6,323.7 m (€6,285.7 m nominal) in 2011 to €8,792.3 m (€9,267.1 m nominal) in 2021 (Supplementary Table S10). The stock value trends at sectoral level generally tracked the equivalent trends in biomass (Figure 5; Supplementary Table S10).

In 2012, the Sdm, Rdf, and DnR_C sectors saw the biggest increase in biomass and stock value compared to 2011. All beef subtypes except beef pedigree and the Sdm and Sbm sectors saw a decrease in stock value in 2014. From 2014 to 2015, the DnR_nC, DnR_C, and Rdf sectors saw the largest year on year increase in sectoral biomass and stock value. Stock values increased in most sectors except the BSY and DRm sectors. All sectors decreased in stock value, apart from DnR_nC. The beef and store sectors had the greatest reduction in biomass in 2016 compared to 2015. In 2021 versus 2020, most sectors except BSB increased in stock value, and biomass declined for the following sectors: BSB, BSY, mixed production, unclassified, DRm, BSY_nR, and BP sectors (Figures 4, 5; Supplementary Tables S9, S10).

### 3.6 Average biomass and average stock value by herd

#### 3.6.1 Herd level annual trends

**Figure 6:**
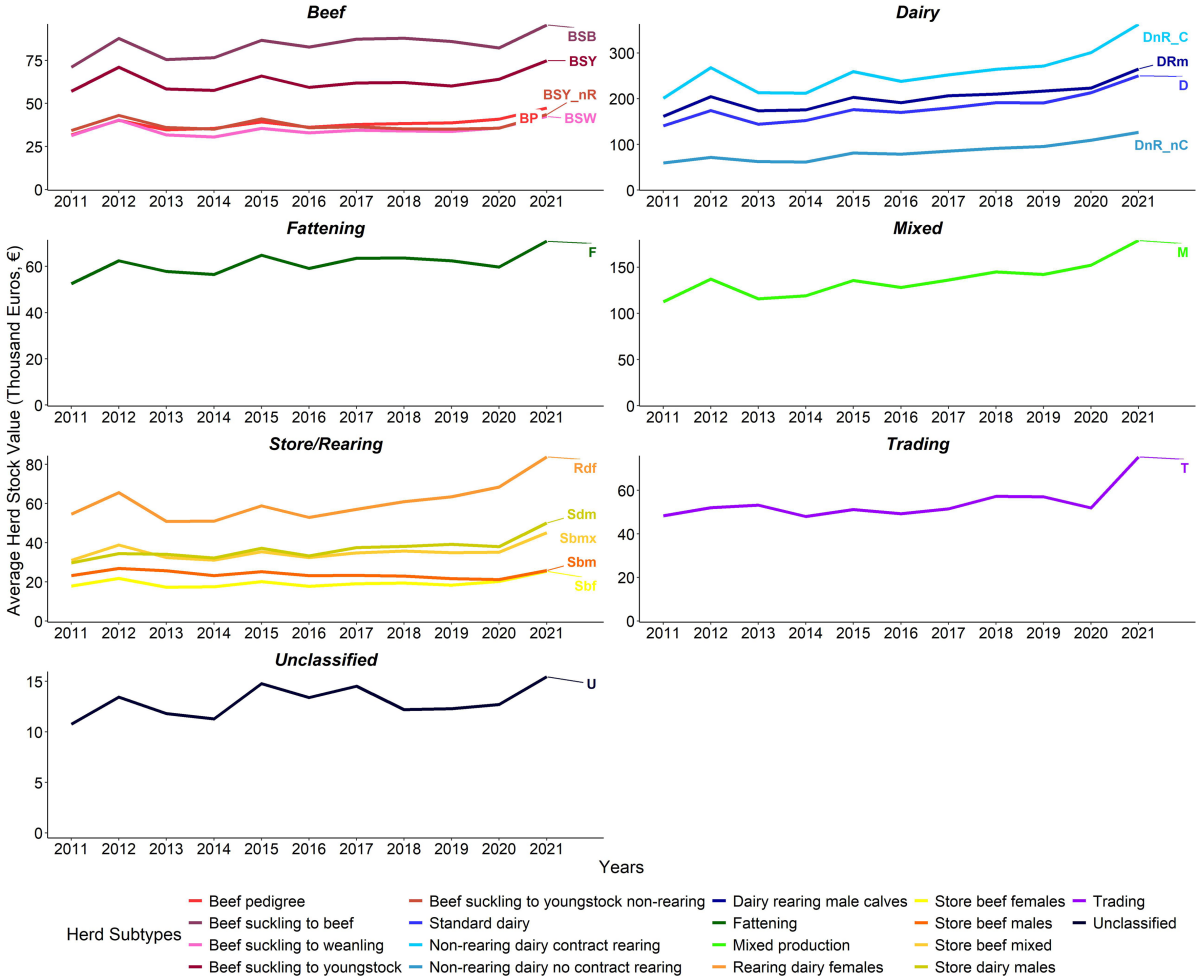
Average herd biomass (‘000 kg) by herd type 2011–2021 (note differences in biomass scales between sectors).

**Figure 7:**
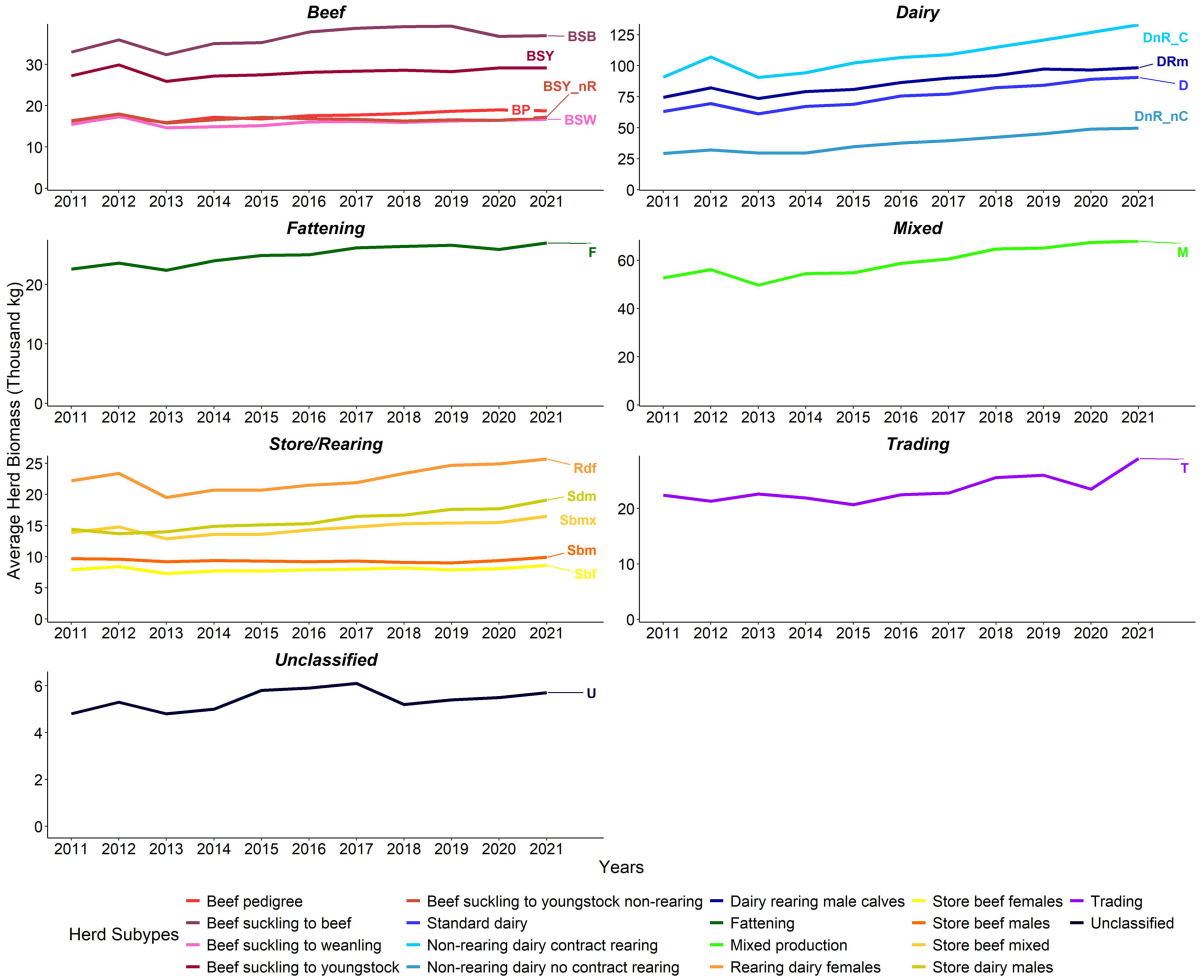
Average herd stock value (€ ‘000) by herd type 2011–2021 (note differences in value scales between sectors). Adjusted for inflation (Base December 2011 = 100) (23).

At herd level, the average biomass and average stock values increased consistently between years from 2011 to 2021 for beef, dairy, fattening, mixed production, and store/rearing herds. In contrast, during the same period unclassified and trading herds’ average herd biomass fluctuated without showing a consistent trend (Figures 6, 7).

2012, 2014, 2016, and 2018 had a considerable year-on-year positive change in the all-cattle herd’s average biomass. The average herd biomass increased in all years except 2013, when it declined for all herd types except Sdm and trading herds (Figure 6; Supplementary Table S11). In 2012, 2015, and 2021, there was a positive year-on-year change in the average herd stock value. The average herd stock value of all herds declined in 2013 and 2016 except for trading herds in 2013 (Figure 7; Supplementary Table S12).

#### 3.6.2 Herd level within-year trends

**Figure 8:**
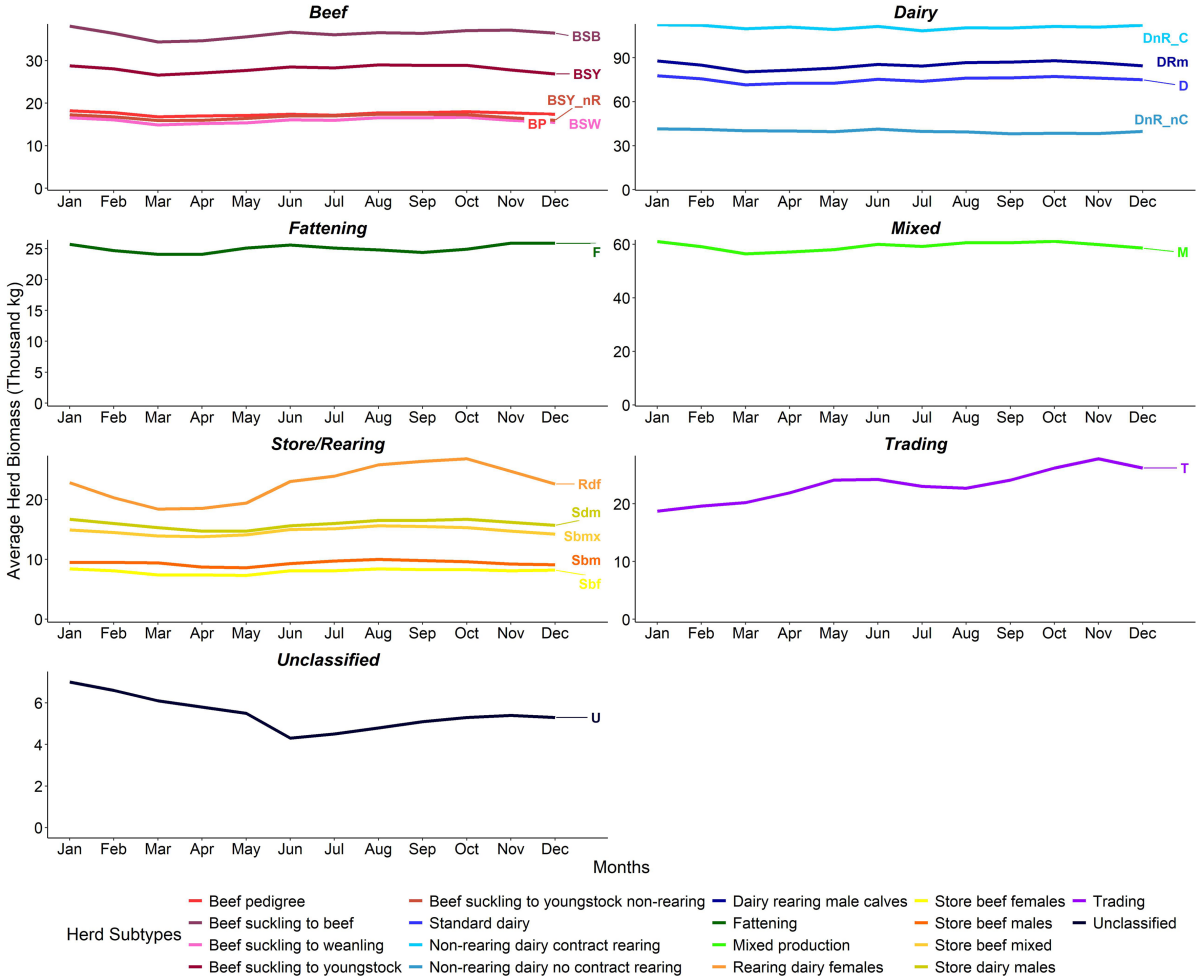
Within-year average herd biomass (‘000 kg) from 2011 to 2021 by herd type (note differences in biomass scales between sectors).

Within year, at herd level, the beef, dairy, mixed production, and fattening herds showed little evidence of seasonality in biomass. Likewise, within the store/rearing sector, Sdm, Sbmx, Sbm, and Sbf herds also showed little evidence of any seasonal patterns. Of the beef herd types, BSB herds had the highest average herd biomass throughout the year, followed by BSY, BP, BSY_nR, and BSW herds. DnR_C had consistently higher herd biomass within year compared to the other dairy herd types. The D and DRm herds were similar within year, with around 10,000 kg of a difference between the two herd subtypes. The lowest herd biomass seen in the dairy herds within-year was the DnR_nC herds (Figure 8).

**Figure 9:**
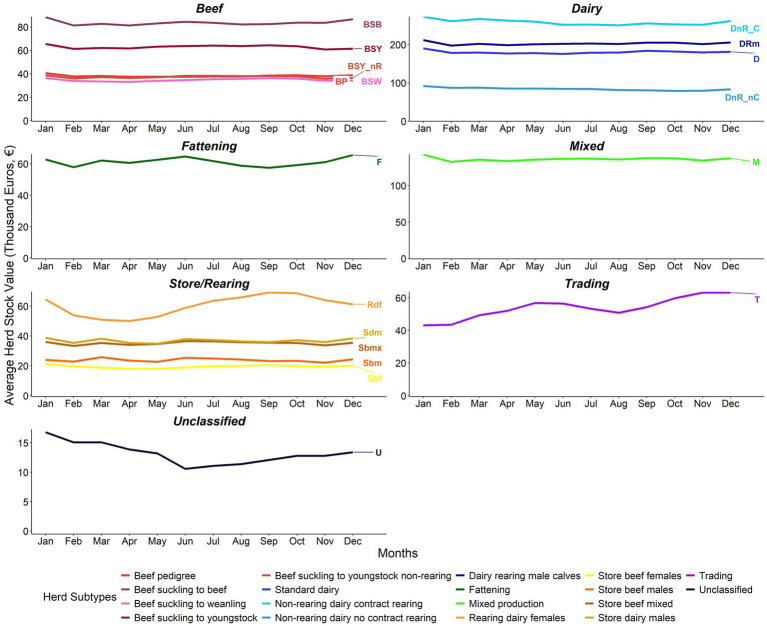
Within-year average herd stock value (€ ‘000) from 2011 to 2021 by herd type (note differences in value scales between sectors). Adjusted for inflation (Base December 2011 = 100) (24).

Sbf and Sbm herds had the lowest herd biomass estimate compared to the other store/rearing herds. The biomass of Rdf herds showed a distinct seasonal pattern, increasing slightly from March to May, before reaching higher values over the summer months and then peaking in October, followed by a sharp decline over the winter. The trading herds also saw a seasonal pattern in biomass, peaking in the spring and autumn months. Estimated stock values for herds followed within-year patterns similar to those for biomass (Figure 9).

### 3.7 Biomass comparison

**Figure 10:**
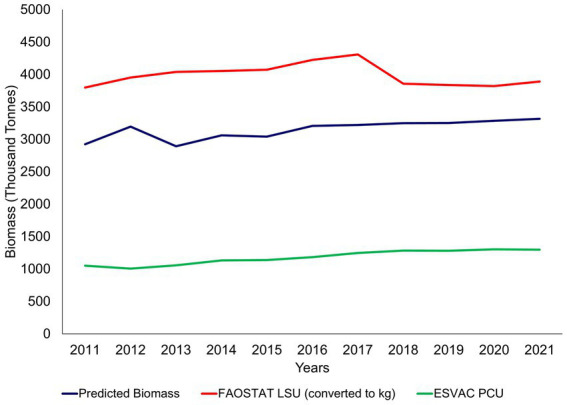
Comparison of biomass metrics (FAOSTAT LSU and ESVAC PCU) to the study’s predicted total biomass estimates (26, 27).

The total biomass estimates of the bovine population in this study were lower than the FAOSTAT LSU when converted to kg and considerably higher than the PCU, as seen in Figure 10 (26, 27).

## 4 Discussion

This paper is the first livestock biomass study in Ireland focusing on national cattle data, including the stock value per herd. Our study takes the entire cattle population into account. Precise livestock numbers are essential when calculating productivity and biomass as inputs to antimicrobial use and climate change calculations (1, 2). In the context of policy discussions on reducing greenhouse gas emissions, water quality issues, antimicrobial usage and other environmental questions, having more accurate estimates of cattle biomass by herd type would support more effective analysis of the challenges to be addressed. For countries where uptake of a movement tracing system is voluntary or non-existent, this approach could be used as an incentive to implement such a system that provides rigorous data for farm reporting, benchmarking, and governmental policy analyses.

Three other studies on livestock biomass and value have previously been conducted in Ethiopia and Indonesia through the Global Burden of Animal Diseases (GBADs) program (11, 12, 29). The GBADs program was launched in 2018 to estimate the animal disease burden of all livestock species and draw comparisons between diseases, production systems, and countries (9, 30, 31). The Indonesian study estimated the biomass of dairy cattle, buffalo, sheep, goats, pigs, and chickens as a whole, and the Ethiopian study estimated the biomass of goats and sheep by sex-age category. Both studies estimated biomass by multiplying the population of each species by the average liveweight (11, 12). Further to this, using the Li, Mayberry (32) approach, the Indonesian study estimated the biomass of beef cattle by multiplying the population number in each age, breed, and sex category by their average liveweight. For equids (horses, donkeys, and mules) in Ethiopia, biomass was calculated using one tropical livestock unit (1 TLU = 250 kg) and multiplying by the population numbers (29). Smith, Ilham (12) calculated the population value by multiplying the biomass estimate by the average price per animal and dividing it by the average liveweight per animal. Jemberu, Li (11), Asteraye, Pinchbeck (29) calculated the population value (also referred to as stock monetary value) by multiplying the total population by the current average market price.

Instead of multiplying by the population numbers, this current study estimated the cattle biomass and stock value at the individual animal level by breed category, age, and sex, using monthly movement data and aggregated that at herd level, summing the herd biomass/value to get the sector biomass/value. This method allowed animals that moved in and out of herds throughout the year to be captured rather than using animal numbers from a single time point in the year. This became apparent when comparing the study’s total biomass to international metrics, such as the LSU and PCU. Both use bovine population numbers that are published on a yearly static basis. The population numbers for the PCU are derived from a Eurostat database (33) whereby the animal population survey is conducted during November/December. The FAOSTAT’s LSU population numbers are derived from an FAOSTAT database (34) grouped yearly at the end of September (35). As the total biomass estimates in this study capture animals throughout the year rather than at a static time, the population numbers differ slightly when compared to the other metrics mentioned above, thus, impacting biomass estimates.

The key variables needed for creating the liveweight and value models were age, sex, breed type, liveweight, market value, month, and year. This information can be gathered at farm-level and national-level through on-farm records and movement and tracing systems, and can be expanded to other species. For countries or researchers that do not have access to such a system or live-updating animal database, following studies that use static animal numbers from publicly accessible databases such as FAOSTAT, expert elicitation and modeling approaches are encouraged, e.g., Jemberu, Li (11), Smith, Ilham (12), Asteraye, Pinchbeck (29), Boeters, Steeneveld (36). This study could expand Ireland’s GHG inventory Tier II data and the inclusion of data from farm advisory tools to get real agricultural activity data, to become Tier III (37). Furthermore, integrating the biomass and value model of this study into profit calculators, farm budget and advisory tools demonstrating predicted liveweights and associated stock value, and with the addition of their own liveweight estimates, could serve as a benchmarking exercise for farmers.

Since 2013, there has been an ongoing decrease in the biomass of the beef sector. However, stock values did not track this and showed a degree of fluctuation from year to year, with most herd types in the beef sector increasing marginally. The concomitant decrease in the beef sector biomass estimates was in contrast to the increased values for the dairy sector. The change in herd numbers and sizes over the years could explain this. The abolition of the EU milk quota in 2015 initiated dairy expansion in Ireland (38). Since then, the number of beef herds has decreased, while the median herd size of beef herds has changed little. However, the median herd size has increased considerably for DnR_C, D, DRm, mixed production, and DnR_nC herds (Supplementary Table S2). Further research is required to determine the key drivers for beef farmers exiting the system. There are many factors that influence the change in value, e.g., structural economic changes, conflict, and economic development, which vary between countries (13). For example, extreme and fluctuating weather impacts production and prices in Europe. Potential policy formation to support farmers’ risk management and adaptation in response to such shocks and possible fodder crises, especially for pasture-based systems. Extreme weather and fodder crises events were highlighted in 2012–2013, 2014–2015, 2017–2018, and 2020 due to prolonged summer droughts and severe winter weather (39).

A previous study by McHugh, Fahey (40) using livestock market data to determine the factors associated with the selling price of cattle in Ireland found that liveweights and growth rates affect price differences, which explains the herd biomass and herd stock value trends in this study being similar between years. When adjusting for liveweight in one of their models, it was found that sex remained significant, suggesting other factors were at play, such as greater potential growth rates in males. Hence, the liveweight and value models were created for females and males separately.

The estimates from the value model are based on the valuations carried out by independent valuers informed by summaries of livestock market prices derived from DS2, which reflect the most up-to-date livestock market values for various categories sold on the open market at that time. A Grant Thornton review of the OFMV Scheme found the Summary Market Prices report used by valuers as a guide during their valuations was robust in its collation of large data, development, analysis, and the outputs reflect the true market value for livestock at a point in time. However, some data may not be captured due to the inability of a departmental representative to attend markets or private sales that happen outside of the open market (41). The value model was validated against the market data, and we found that the predicted values provided a good match to the real-world market values. In the validation process, the value estimates from the market data could not be compared with the predicted estimates from the value model using direct age data due to the lack of date of birth information in the market dataset. For each animal category in the market data, an age range in months was given for each animal category (lactation stage, calf, etc.). This allowed for the market data summary estimates to be matched with the predicted summary estimates using the year, age/animal category, breed, and sex information. Comparing the value estimates with the factory prices will add robustness to the results and validation process. As mentioned above, this study focused on stock value in terms of liveweight; however, there are other aspects to consider, such as production outputs and total economic value (13).

Biomass was not validated due to the lack of a comparator. The liveweights used to create the herd biomass estimates were derived from the bTB reactor animals and may not fully reflect the true liveweights among the Irish cattle population. This is due to two possible factors. Firstly, the population of bTB-infected cattle may have a growth rate different from that of non-infected animals in the period between infection and detection, possibly lower weight gain (42). Secondly, the population of infected cattle is not randomly distributed among the total cattle population; while our model accounted for the individual animal-level characteristics, it was not possible to account for other variables such as genetic susceptibility to bTB infection and risk factors at animal and herd level which affect bTB risk in the Irish context (43).

Additionally, the use of DS1 data for the liveweight and value models may introduce sampling bias, where animals in high-risk areas and production systems are more likely to be captured in the data, however, our process in running the models through DS3 to provide an estimate for every animal present in the country monthly based on breed type, age, and sex, representative to the national herd. In contrast, our biomass estimates assume that all animals in the population are healthy. As a result, these estimates do not reflect the true national picture as they assume no disease outbreaks or diseased animals are in the population. For example, bovine viral diarrhea (BVD) causes a loss of productivity in infected calves, infertility of cows, and premature culling (44, 45). However, the estimates from this study do provide a baseline for comparing the ‘healthy’ estimates with the ‘diseased’ estimates to give an animal health loss estimate, contributing to the Animal Health Loss Envelope (AHLE) (10).

The estimates from this study must be considered alongside overall farm income and expenditure. The herd stock value does not reflect the total economic value of the herd as direct costs (e.g., housing, labor, feedstock, veterinary costs) are not deducted from the value, and the addition of direct payments (government subsidies) or productive outputs such as milk yields are not included. For example, Hennessy, Doran (46) found that, on average, the beef sector relies on direct payments as income, as the production costs exceed market prices.

## 5 Conclusion

The utility of precise and accurate biomass and stock value estimates is that they can be used as a basis for better assessments of profitability, animal health losses, environmental emissions, the use of antimicrobials in cattle and trends in biomass and value over time.

Our estimates were aggregated by herd type to allow for a granular view of Ireland’s different cattle production systems. The results from this study can be used to improve the economic efficiency and productivity of the cattle sector as a whole and at herd level by estimating the optimum biomass and value for each animal given its age, breed category, and sex. The study demonstrates how existing traceability and disease program databases can be used to generate better estimates of biomass and stock value, and these methods are applicable to other countries that have similar animal identification systems.

Although the biomass and stock value can be used as they are now, spatial analysis is recommended to gain more insight into the trends described in this study and highlight key areas in the country that may have high or low levels of livestock biomass. More valuation data from private sales, factory, and slaughter prices are needed to continue strengthening the value model. More liveweight information, possibly from farming records and advisory tools, is needed to continue strengthening the biomass estimates. Further research is needed into the distribution of biomass along the food value chain.

## Data Availability

The datasets presented in this study can be found in online repositories. The names of the repository/repositories and accession number(s) can be found at: https://github.com/ejbamurray/biomass_value_Ireland.
