# Supplementary material for: Estimating sectoral livestock biomass and stock value using data from national diseases eradication programs: a case study based on the Irish cattle herd from 2011 to 2021
Source: Front Vet Sci. 2025 Oct 2;12:1648948. doi: 10.3389/fvets.2025.1648948 (PMC12529700; doi:10.3389/fvets.2025.1648948)
Supplement: Supplementary file 1 [file Supplementary_file_1.docx]

Supplementary Material

Supplementary Table S1. The breeds of the animal records in this study are characterized by breed category: British-Irish beef, Continental beef, and dairy. The breed category was determined using a breed list managed by the Department of Agriculture, Food, and the Marine (18).

| **British-Irish Beef** | | **Continental Beef** | | **Dairy** | |
| --- | --- | --- | --- | --- | --- |
| **Code** | **Breed Name** | **Code** | **Breed Name** | **Code** | **Breed Name** |
| AA | Angus | AK | Ankole | AM | Armoricaine |
| BG | Blue Grey | AL | Australian Lowline | AN | Angler |
| BY | Belted Galloway | AU | Aubrac | AY | Ayrshire |
| DE | Devon | BA | Blonde D'Aquitane | BL | Blue Albion |
| DN | Droimeann | BB | Belgian Blue | BP | Bretonne Pie-Noire |
| DX | Dexter | BE | Beefalo | BS | Brown Swiss |
| EP | English Park | BF | Water Buffalo | FE | Frisona Espagnola |
| GA | Galloway | BI | Bison | FR | Holstein/Freisian |
| GL | Gloucester | BR | Brahman | GB | Groninger Blaarkop |
| GS | Murray Grey | BT | Baltata Romaneasca | GU | Guernsey |
| HE | Hereford | BW | British White | JE | Jersey |
| HI | Highland | BZ | Bazadais | LJ | Lithuanian Black and White |
| IM | Irish Maol | CB | Canadian Black | MK | Malkekorthorn |
| KE | Kerry | CH | Charolais | MO | Montbeliarde |
| OE | Old English | CI | Chianina | MS | Blended Milking Shorthorn |
| SD | South Devon | CL | Chillingham | MY | Mri/Mry |
| SH | Shorthorn | EF | East Finnish Brown | NO | Normande |
| SL | Shetland | GC | Gasconne | NR | Norwegian Red |
| SU | Sussex | GV | Gelbvieh | RB | Rotbunte |
| TY | Tyrone Black | GY | Gayal | RD | Danish Red |
| WB | Welsh Black | IN | Inra 95 | RE | Reggiana |
| WG | White Galloway | LH | Longhorn | SR | Swedish Red |
|  |  | LM | Limousin |  |  |
|  |  | LR | Lincoln Red |  |  |
|  |  | LU | Luing |  |  |
|  |  | LV | Lakenvelder |  |  |
|  |  | MA | Maine Anjou |  |  |
|  |  | MH | Marchigiana |  |  |
|  |  | PI | Piedmontese |  |  |
|  |  | PS | Pustertaler Sprinzen |  |  |
|  |  | PT | Partenaise |  |  |
|  |  | PZ | Pinzgauer |  |  |
|  |  | RM | Romagnola |  |  |
|  |  | RP | Red Poll |  |  |
|  |  | SA | Salers |  |  |
|  |  | SG | Swiss Grey |  |  |
|  |  | SI | Simmental |  |  |
|  |  | SP | Speckle Park |  |  |
|  |  | ST | Stabiliser |  |  |
|  |  | TT | Tarantaise-Tarina |  |  |
|  |  | VA | Vaynol |  |  |
|  |  | VB | Verbeterd Roodbont Vleesras |  |  |
|  |  | VO | Vosgienne |  |  |
|  |  | WA | Wagyu |  |  |
|  |  | WP | White Park |  |  |
|  |  | WW | Welsh White |  |  |
|  |  | YK | Yak |  |  |
|  |  | ZE | Zebu |  |  |

Supplementary Table S2. Animal categories and their age range for the validation of the value model (DS2).

| **Dairy** | |
| --- | --- |
| **Animal Class** | **Age Range (Months)** |
| Heifer Calf | 1-3 |
| Weaned Heifer | 4-14 |
| Maiden Heifer | 15-18 |
| In-calf Heifer | 19-23 |
| Lactation 1 | 24-35 |
| Lactation 2 | 36-47 |
| Lactation 3 | 48-59 |
| Lactation 4+ | 60-141 |
| **Beef** | |
| **Animal Class** | **Age Range (Months)** |
| Calf | 1-6 |
| Weaned | 7-12 |
| In-calf Heifer | 13-36 |
| Young Bull Feeder | 13-16 |
| Light Store | 13-18 |
| Forward Store | 19-30 |
| Parity 1 | 24-35 |
| Parity 2 | 36-47 |
| Parity 3 | 48-59 |
| Parity 4+ | 60-141 |
| Finished | 17-30 |
| Cull Cow | 3-33 |


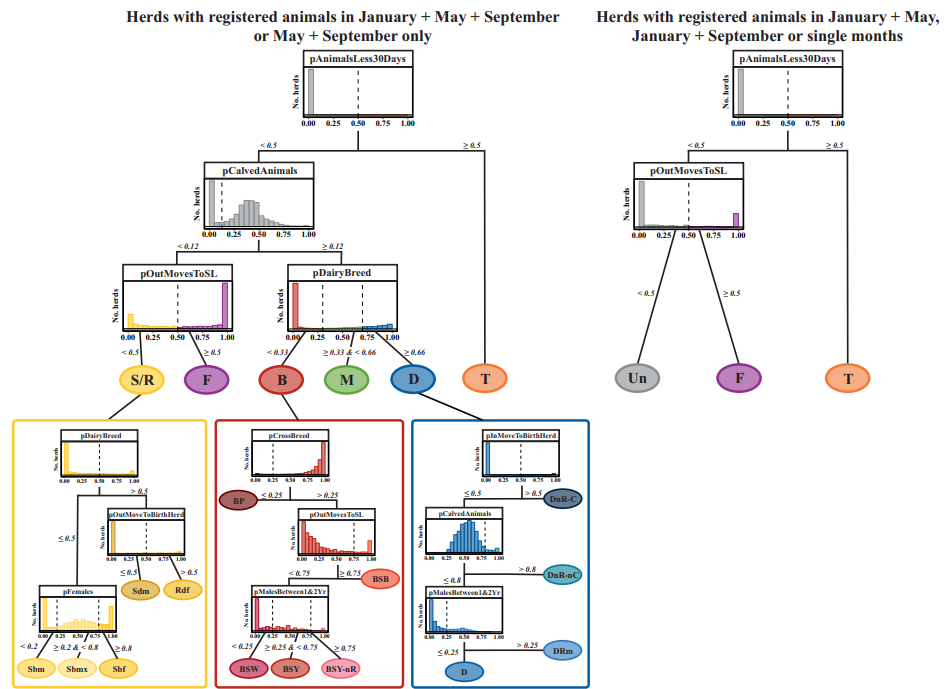


Supplementary Figure S1. Modified decision tree for herd classification based on the Brock, Lange (22) herd classification model.

Supplementary Table S3. Herd numbers (%) by a) main herd type and b) herd subtype from 2011 to 2021.

|  | **2011** | **2012** | **2013** | **2014** | **2015** | **2016** | **2017** | **2018** | **2019** | **2020** | **2021** |
| --- | --- | --- | --- | --- | --- | --- | --- | --- | --- | --- | --- |
| **Beefᵃ** | 60787 (53.5) | 60146 (52.9) | 59899 (52.7) | 58307 (51.3) | 56920 (50.1) | 56029 (49.7) | 54889 (49.3) | 53980 (49.1) | 52394 (48.2) | 51401 (47.3) | 49659 (45.7) |
| *Beef Pedigree Herds*ᵇ | 2458 (2.3) | 2279 (2.1) | 2201 (2.0) | 2211 (2.0) | 2198 (2.0) | 2166 (2.0) | 2142 (2.0) | 2193 (2.0) | 2233 (2.1) | 2206 (2.0) | 2211 (2.0) |
| *Beef Suckling to Beef*ᵇ | 7294 (6.7) | 6891 (6.3) | 7201 (6.6) | 7074 (6.5) | 6407 (5.9) | 6554 (6.0) | 6183 (5.7) | 6421 (5.9) | 6231 (5.7) | 6012 (5.5) | 5031 (4.6) |
| *Beef Suckling to Weanlings*ᵇ | 31864 (29.3) | 33161 (30.5) | 29139 (26.8) | 28291 (26) | 30539 (28.1) | 29469 (27.1) | 29279 (26.9) | 28220 (26) | 27519 (25.3) | 26134 (24.1) | 26636 (24.5) |
| *Beef Suckling to Youngstock*ᵇ | 14974 (13.8) | 13827 (12.7) | 17082 (15.7) | 16405 (15.1) | 13835 (12.7) | 14182 (13.1) | 13738 (12.6) | 13808 (12.7) | 13145 (12.1) | 13739 (12.6) | 12649 (11.6) |
| *Beef Suckling to Youngstock non-rearing*ᵇ | 4197 (3.9) | 3988 (3.7) | 4276 (3.9) | 4326 (4.0) | 3941 (3.6) | 3658 (3.4) | 3547 (3.3) | 3338 (3.1) | 3266 (3.0) | 3310 (3.0) | 3132 (2.9) |
| **Dairyᵃ** | 13274 (11.7) | 13747 (12.1) | 13748 (12.1) | 13706 (12.1) | 13482 (11.9) | 13455 (11.9) | 12995 (11.7) | 12474 (11.3) | 12437 (11.4) | 12269 (11.3) | 12524 (11.5) |
| *Standard Dairy Herd*ᵇ | 8337 (7.7) | 8516 (7.8) | 7588 (7) | 7818 (7.2) | 8163 (7.5) | 7987 (7.4) | 7794 (7.2) | 7310 (6.7) | 7445 (6.9) | 7329 (6.7) | 7698 (7.1) |
| *Non Rearing Dairy - Contract Rearing*ᵇ | 545 (0.5) | 654 (0.6) | 720 (0.7) | 833 (0.8) | 914 (0.8) | 932 (0.9) | 929 (0.9) | 929 (0.9) | 1056 (1.0) | 1081 (1.0) | 1100 (1.0) |
| *Non Rearing Dairy - No Contract Rearing*ᵇ | 558 (0.5) | 564 (0.5) | 506 (0.5) | 556 (0.5) | 685 (0.6) | 770 (0.7) | 807 (0.7) | 900 (0.8) | 946 (0.9) | 985 (0.9) | 1004 (0.9) |
| *Dairy Herd Rearing Male Calves*ᵇ | 3834 (3.5) | 4013 (3.7) | 4934 (4.5) | 4499 (4.1) | 3720 (3.4) | 3766 (3.5) | 3465 (3.2) | 3335 (3.1) | 2990 (2.8) | 2874 (2.6) | 2722 (2.5) |
| **Fatteningᵃ** | 16585 (14.6) | 15166 (13.4) | 15581 (13.7) | 16624 (14.6) | 15730 (13.8) | 16454 (14.6) | 16981 (15.2) | 17055 (15.5) | 17273 (15.9) | 17280 (15.9) | 16903 (15.6) |
| **Mixed Productionᵃ** | 5227 (4.6) | 5234 (4.6) | 5171 (4.6) | 5100 (4.5) | 5285 (4.7) | 5349 (4.7) | 5330 (4.8) | 5435 (4.9) | 5150 (4.7) | 5315 (4.9) | 5024 (4.6) |
| **Store/Rearingᵃ** | 12360 (10.9) | 14640 (12.9) | 14542 (12.8) | 15219 (13.4) | 16415 (14.5) | 16333 (14.5) | 16606 (14.9) | 16323 (14.8) | 17072 (15.7) | 17044 (15.7) | 19388 (17.8) |
| *Rearing Dairy Females*ᵇ | 177 (0.2) | 259 (0.2) | 291 (0.3) | 362 (0.3) | 416 (0.4) | 414 (0.4) | 439 (0.4) | 413 (0.4) | 516 (0.5) | 562 (0.5) | 561 (0.5) |
| *Store - Beef Females*ᵇ | 3421 (3.1) | 3727 (3.4) | 3521 (3.2) | 3906 (3.6) | 4235 (3.9) | 4066 (3.7) | 4253 (3.9) | 4354 (4.0) | 4728 (4.4) | 4538 (4.2) | 4971 (4.6) |
| *Store - Beef Males*ᵇ | 4777 (4.4) | 5433 (5.0) | 5287 (4.9) | 5435 (5.0) | 5762 (5.3) | 5550 (5.1) | 5683 (5.2) | 5771 (5.3) | 6060 (5.6) | 5789 (5.3) | 6565 (6.0) |
| *Store - Beef Mixed*ᵇ | 2398 (2.2) | 2424 (2.2) | 2570 (2.4) | 2803 (2.6) | 3146 (2.9) | 3216 (3.0) | 3426 (3.2) | 3442 (3.2) | 3472 (3.2) | 3782 (3.5) | 4408 (4.1) |
| *Store - Dairy Males*ᵇ | 1587 (1.5) | 2797 (2.6) | 2873 (2.6) | 2713 (2.5) | 2856 (2.6) | 3087 (2.8) | 2805 (2.6) | 2343 (2.2) | 2296 (2.1) | 2373 (2.2) | 2883 (2.7) |
| **Trader (Dealer)ᵃ** | 1010 (0.9) | 1070 (0.9) | 1089 (1.0) | 1008 (0.9) | 1038 (0.9) | 981 (0.9) | 929 (0.8) | 870 (0.8) | 851 (0.8) | 890 (0.8) | 830 (0.8) |
| **Unclassifiedᵃ** | 5771 (5.1) | 4871 (4.3) | 4367 (3.8) | 3731 (3.3) | 4726 (4.2) | 4069 (3.6) | 3676 (3.3) | 3783 (3.4) | 3517 (3.2) | 3945 (3.6) | 3736 (3.4) |
| **Total** | **115014 (100)** | **114824 (100)** | **114397 (100)** | **113695 (100)** | **113596 (100)** | **112670 (100)** | **111406 (100)** | **109920 (100)** | **108659 (100)** | **108144 (100)** | **108064 (100)** |

Supplementary Table S4. Median herd size (range) by a) main herd type and b) herd subtype from 2011 to 2021.

|  | **2011** | **2012** | **2013** | **2014** | **2015** | **2016** | **2017** | **2018** | **2019** | **2020** | **2021** |
| --- | --- | --- | --- | --- | --- | --- | --- | --- | --- | --- | --- |
| **Beefᵃ** | 29 (1-2437) | 33 (1-1501) | 31 (1-1363) | 30 (1-1943) | 30 (1-1568) | 31 (1-3366) | 32 (1-4089) | 31 (1-2793) | 31 (1-2476) | 32 (1-1729) | 32 (1-1788) |
| *Beef Pedigree Herds*ᵇ | 19 (1-436) | 22 (1-622) | 21 (1-429) | 22 (1-505) | 22 (1-456) | 23 (1-440) | 24 (1-403) | 24 (1-454) | 25 (1-2250) | 25 (1-514) | 25 (1-573) |
| *Beef Suckling to Beef*ᵇ | 48 (1-2437) | 53 (1-1497) | 50 (1-1363) | 52 (1-1943) | 54 (1-1568) | 58 (1-3366) | 60 (1-4089) | 60 (1-2793) | 61 (1-2476) | 55 (1-1729) | 56 (1-1389) |
| *Beef Suckling to Weanlings*ᵇ | 22 (1-761) | 26 (1-1387) | 22 (1-1092) | 22 (1-765) | 23 (1-685) | 23 (1-1029) | 24 (1-974) | 23 (1-1151) | 23 (1-1028) | 24 (1-957) | 24 (1-1788) |
| *Beef Suckling to Youngstock*ᵇ | 45 (1-1434) | 50 (1-1501) | 45 (1-819) | 45 (1-1004) | 47 (1-980) | 47 (1-1042) | 47 (1-1079) | 47 (1-952) | 46 (1-731) | 47 (1-808) | 47 (1-803) |
| *Beef Suckling to Youngstock non-rearing*ᵇ | 25 (1-647) | 28 (1-719) | 26 (1-668) | 25 (1-655) | 27 (1-396) | 26 (1-617) | 26 (1-423) | 24 (1-449) | 24 (1-398) | 25 (1-477) | 26 (1-422) |
| **Dairyᵃ** | 120 (1-1948) | 134 (1-2776) | 128 (1-1997) | 130 (1-2138) | 133 (1-2196) | 138 (1-2195) | 143 (1-2337) | 147 (1-2228) | 149 (1-2286) | 152 (1-2300) | 154 (1-2388) |
| *Standard Dairy Herd*ᵇ | 112 (1-1590) | 124 (1-2776) | 115 (1-1997) | 120 (1-2138) | 125 (1-2196) | 131 (1-2195) | 135 (1-2266) | 140 (1-2215) | 143 (1-2109) | 149 (1-2300) | 152 (1-2388) |
| *Non Rearing Dairy - Contract Rearing*ᵇ | 161 (3-858) | 187 (3-2425) | 174 (3-1324) | 169 (17-1602) | 179 (1-1403) | 180 (1-1728) | 186 (1-1898) | 193 (1-1871) | 195 (1-1853) | 200 (1-1801) | 210 (3-2141) |
| *Non Rearing Dairy - No Contract Rearing*ᵇ | 38 (1-611) | 39 (1-875) | 40 (1-1001) | 37 (1-705) | 48 (1-879) | 48 (1-817) | 49 (1-848) | 54 (1-1104) | 57 (1-1107) | 61 (1-1265) | 62 (1-1340) |
| *Dairy Herd Rearing Male Calves*ᵇ | 148 (1-1948) | 165 (1-2424) | 158 (1-1597) | 157 (1-1708) | 162 (1-1779) | 167 (1-1878) | 177 (1-2337) | 178 (2-2228) | 183 (1-2286) | 175 (1-1804) | 177 (1-1731) |
| **Fatteningᵃ** | 27 (1-2194) | 30 (1-2886) | 30 (1-2640) | 30 (1-3688) | 31 (1-4092) | 32 (1-3762) | 33 (1-3687) | 33 (1-4248) | 33 (1-4103) | 32 (1-3886) | 34 (1-3985) |
| **Mixed Productionᵃ** | 97 (1-2660) | 104 (1-2935) | 97 (1-1266) | 100 (1-1245) | 104 (1-1704) | 109 (1-1239) | 115 (1-1174) | 121 (1-1383) | 120 (1-1516) | 124 (1-1514) | 123 (1-1465) |
| **Store/Rearingᵃ** | 16 (1-1610) | 17 (1-1196) | 16 (1-978) | 16 (1-1173) | 17 (1-842) | 18 (1-1237) | 18 (1-4042) | 18 (1-3749) | 17 (1-1097) | 18 (1-3207) | 20 (1-2335) |
| *Rearing Dairy Females*ᵇ | 46 (1-286) | 47 (1-532) | 37 (1-485) | 37 (1-692) | 40 (1-342) | 41 (1-357) | 42 (1-453) | 45 (1-579) | 47 (1-792) | 50 (1-900) | 51 (1-1280) |
| *Store - Beef Females*ᵇ | 12 (1-616) | 13 (1-495) | 12 (1-955) | 12 (1-657) | 13 (1-842) | 12 (1-784) | 13 (1-638) | 12 (1-898) | 12 (1-861) | 13 (1-803) | 14 (1-753) |
| *Store - Beef Males*ᵇ | 14 (1-700) | 15 (1-713) | 14 (1-633) | 14 (1-1013) | 14 (1-838) | 14 (1-938) | 14 (1-4042) | 14 (1-3749) | 14 (1-1097) | 15 (1-1425) | 15 (1-698) |
| *Store - Beef Mixed*ᵇ | 23 (1-1610) | 25 (1-1196) | 23 (1-433) | 23 (1-1140) | 25 (1-695) | 26 (1-1237) | 27 (1-1177) | 27 (1-1214) | 27 (1-1051) | 28 (1-3207) | 30 (1-2335) |
| *Store - Dairy Males*ᵇ | 24 (1-704) | 24 (1-1032) | 24 (1-978) | 23 (1-1173) | 25 (1-719) | 26 (1-1006) | 28 (1-3005) | 29 (1-792) | 29 (1-865) | 30 (1-889) | 33 (1-1530) |
| **Trader (Dealer)ᵃ** | 21 (1-2129) | 23 (1-950) | 25 (1-1853) | 22 (1-1904) | 21 (1-1550) | 22 (1-4099) | 22 (1-2651) | 22 (1-20078) | 21 (1-17936) | 22 (1-2329) | 27 (1-2775) |
| **Unclassifiedᵃ** | 6 (1-511) | 7 (1-725) | 7 (1-840) | 6 (1-691) | 7 (1-926) | 7 (1-1747) | 7 (1-5779) | 7 (1-647) | 7 (1-1444) | 7 (1-1084) | 7 (1-529) |

Supplementary Table S5. Estimated coefficients from the female liveweight (kg) model.

| **Variable** | **Category** | **Estimate (95% confidence intervals)** | **P-value** |
| --- | --- | --- | --- |
| **Intercept** |  | 91.38 (65.94, 116.82) | <0.001 |
| **Breed category** | Beef continental | Referent | <0.001 |
|  | Beef traditional | -37.25 (-47.74, -26.76) | <0.001 |
|  | Dairy traditional | -25.95 (-35.86, -16.05) | <0.001 |
| **Month** | January | Referent | <0.001 |
|  | February | -10 (-32.37, 12.37) | 0.381 |
|  | March | -29.52 (-51.95, -7.09) | 0.010 |
|  | April | -23.35 (-44.76, -1.94) | 0.033 |
|  | May | -3.53 (-24.48, 17.43) | 0.742 |
|  | June | 2.21 (-18.45, 22.87) | 0.834 |
|  | July | -6.13 (-26.78, 14.53) | 0.561 |
|  | August | 13.59 (-7.03, 34.21) | 0.197 |
|  | September | 14.15 (-6.05, 34.36) | 0.170 |
|  | October | 13.13 (-7.57, 33.84) | 0.214 |
|  | November | 3.93 (-17.06, 24.93) | 0.714 |
|  | December | -8.93 (-30.72, 12.87) | 0.422 |
| **Year** | 2011 | Referent | <0.001 |
|  | 2012 | 11.26 (-8.95, 31.47) | 0.275 |
|  | 2013 | -1.79 (-23.09, 19.5) | 0.869 |
|  | 2014 | 0.9 (-18.39, 20.2) | 0.927 |
|  | 2015 | 20.11 (0.15, 40.07) | 0.048 |
|  | 2016 | 16.82 (-2.8, 36.44) | 0.093 |
|  | 2017 | 2.77 (-16.8, 22.34) | 0.782 |
|  | 2018 | 1.35 (-18.69, 21.4) | 0.895 |
|  | 2019 | 5.86 (-13.52, 25.24) | 0.554 |
|  | 2020 | 24.39 (5.74, 43.04) | 0.011 |
|  | 2021 | 7.33 (-12.52, 27.18) | 0.469 |
|  | 2022 | 66.68 (41.88, 91.47) | <0.001 |
| **Age (days)** |  | 0.7 (0.67, 0.74) | <0.001 |

Supplementary Table S6. Estimated coefficients from the female value (€) model.

| **Variable** | **Category** | **Estimate (95% confidence intervals)** | **P-value** |
| --- | --- | --- | --- |
| **Intercept** |  | 316.09 (252.84, 379.33) | <0.001 |
| **Breed category** | Beef continental | Referent | <0.001 |
|  | Beef traditional | -211.25 (-242.45, -180.06) | <0.001 |
|  | Dairy traditional | 5.55 (-16.55, 27.64) | 0.623 |
| **Month** | January | Referent | <0.001 |
|  | February | -44.04 (-103.81, 15.72) | 0.149 |
|  | March | 4.76 (-54.51, 64.02) | 0.875 |
|  | April | -6.82 (-63.69, 50.05) | 0.814 |
|  | May | 6.25 (-49.64, 62.14) | 0.827 |
|  | June | -24.28 (-79.46, 30.9) | 0.389 |
|  | July | 11.78 (-42.27, 65.83) | 0.669 |
|  | August | 7.12 (-46.19, 60.43) | 0.794 |
|  | September | 23.44 (-29.18, 76.06) | 0.383 |
|  | October | -2.1 (-56.23, 52.02) | 0.939 |
|  | November | 6.22 (-48.4, 60.85) | 0.823 |
|  | December | 33.78 (-23.57, 91.14) | 0.248 |
| **Year** | 2011 | Referent | <0.001 |
|  | 2012 | 86.43 (38.86, 134.01) | <0.001 |
|  | 2013 | 24.08 (-27.33, 75.49) | 0.359 |
|  | 2014 | -31.47 (-79.23, 16.29) | 0.197 |
|  | 2015 | 114.94 (64.65, 165.23) | <0.001 |
|  | 2016 | 31.33 (-17.11, 79.77) | 0.205 |
|  | 2017 | 34.29 (-12.88, 81.46) | 0.154 |
|  | 2018 | 104.77 (57.34, 152.2) | <0.001 |
|  | 2019 | 63.59 (16.76, 110.42) | 0.008 |
|  | 2020 | 126.13 (80.25, 172.01) | <0.001 |
|  | 2021 | 395.2 (347.95, 442.46) | <0.001 |
|  | 2022 | 395.86 (326.25, 465.47) | <0.001 |
| **Age (days)** |  | 1.43 (1.36, 1.5) | <0.001 |

Supplementary Table S7. Estimated coefficients from the male liveweight (kg) model.

| **Variable** | **Category** | **Estimate (95% confidence intervals)** | **P-value** |
| --- | --- | --- | --- |
| **Intercept** |  | 95.87 (58.6, 133.13) | <0.001 |
| **Breed category** | Beef continental | Referent | <0.001 |
|  | Beef traditional | -43.24 (-57.6, -28.87) | <0.001 |
|  | Dairy traditional | -45.84 (-61.61, -30.07) | <0.001 |
| **Month** | January | Referent | <0.001 |
|  | February | 7.41 (-26.22, 41.03) | 0.666 |
|  | March | -5.73 (-39.72, 28.27) | 0.741 |
|  | April | -17.59 (-49.7, 14.51) | 0.283 |
|  | May | -7.39 (-39.36, 24.58) | 0.651 |
|  | June | 4.81 (-27.69, 37.31) | 0.772 |
|  | July | 6.03 (-25.13, 37.2) | 0.705 |
|  | August | 18.34 (-13.32, 50) | 0.256 |
|  | September | 34.29 (3.39, 65.2) | 0.030 |
|  | October | 30.18 (-2.21, 62.58) | 0.068 |
|  | November | 10.01 (-22.71, 42.74) | 0.549 |
|  | December | -4.22 (-39.76, 31.32) | 0.816 |
| **Year** | 2011 | Referent | <0.001 |
|  | 2012 | 11.85 (-14.41, 38.11) | 0.377 |
|  | 2013 | -17.4 (-44.58, 9.79) | 0.210 |
|  | 2014 | -0.15 (-26.03, 25.74) | 0.991 |
|  | 2015 | 1.87 (-25.58, 29.31) | 0.894 |
|  | 2016 | 12.28 (-14.96, 39.52) | 0.377 |
|  | 2017 | 23.25 (-5.07, 51.57) | 0.108 |
|  | 2018 | 11.94 (-16.51, 40.38) | 0.411 |
|  | 2019 | 10.48 (-19.43, 40.39) | 0.492 |
|  | 2020 | 22.17 (-7.69, 52.03) | 0.146 |
|  | 2021 | 38.65 (10.01, 67.28) | 0.008 |
|  | 2022 | 47.56 (3.1, 92.01) | 0.036 |
| **Age (days)** |  | 0.71 (0.66, 0.75) | <0.001 |

Supplementary Table S8. Estimated coefficients from the male value (€) model.

| **Variable** | **Category** | **Estimate (95% confidence intervals)** | **P-value** |
| --- | --- | --- | --- |
| **Intercept** |  | 281.34 (150, 412.68) | <0.001 |
| **Breed category** | Beef continental | Referent | <0.001 |
|  | Beef traditional | -229.62 (-275.44, -183.8) | <0.001 |
|  | Dairy traditional | -348.11 (-396.86, -299.36) | <0.001 |
| **Month** | January | Referent | <0.001 |
|  | February | -11.7 (-117.01, 93.61) | 0.828 |
|  | March | 96.12 (-7.21, 199.45) | 0.068 |
|  | April | 30.38 (-73.18, 133.93) | 0.565 |
|  | May | 45.7 (-54.98, 146.38) | 0.374 |
|  | June | 137.82 (36.27, 239.36) | 0.008 |
|  | July | 94.56 (-4.57, 193.69) | 0.062 |
|  | August | 55.71 (-45.41, 156.84) | 0.280 |
|  | September | 1.47 (-98.85, 101.79) | 0.977 |
|  | October | 79.66 (-19.62, 178.94) | 0.116 |
|  | November | -4.86 (-105.61, 95.89) | 0.925 |
|  | December | 91.51 (-16.84, 199.85) | 0.098 |
| **Year** | 2011 | Referent | <0.001 |
|  | 2012 | 98.28 (22.12, 174.43) | 0.012 |
|  | 2013 | 54.15 (-25.23, 133.54) | 0.181 |
|  | 2014 | -15.92 (-95.24, 63.39) | 0.694 |
|  | 2015 | 82.31 (-0.72, 165.33) | 0.052 |
|  | 2016 | 95.61 (13.15, 178.07) | 0.023 |
|  | 2017 | 65.58 (-22.66, 153.81) | 0.145 |
|  | 2018 | -2.2 (-89.76, 85.37) | 0.961 |
|  | 2019 | 89.67 (-3.55, 182.89) | 0.060 |
|  | 2020 | -48.01 (-139.05, 43.03) | 0.301 |
|  | 2021 | 183.96 (93.72, 274.21) | <0.001 |
|  | 2022 | 321.14 (186.39, 455.88) | <0.001 |
| **Age (days)** |  | 2.00 (1.35, 2.66) | <0.001 |

Supplementary Table S9. Sector biomass ('000 tonnes) by a) main herd type and b) herd subtype from 2011 to 2021.

| **Herd Classes** | **2011** | **2012** | **2013** | **2014** | **2015** | **2016** | **2017** | **2018** | **2019** | **2020** | **2021** |
| --- | --- | --- | --- | --- | --- | --- | --- | --- | --- | --- | --- |
| **Beefᵃ** | 1,246.3 | 1,350.5 | 1,203.2 | 1,224.7 | 1,175.2 | 1,217.8 | 1,199.4 | 1,190.9 | 1,159.5 | 1,146.3 | 1,093.1 |
| *Beef Pedigree*ᵇ | 38.1 | 39.4 | 34.9 | 37.9 | 36.8 | 38.1 | 38.0 | 39.6 | 41.7 | 41.8 | 41.3 |
| *Beef Suckling to Beef*ᵇ | 239.8 | 247.2 | 232.7 | 247.5 | 225.8 | 247.6 | 238.9 | 250.9 | 244.0 | 220.4 | 185.2 |
| *Beef Suckling to Weanlings*ᵇ | 491.4 | 579.3 | 425.8 | 421.2 | 463.9 | 472.7 | 473.7 | 451.7 | 447.2 | 429.0 | 443.9 |
| *Beef Suckling to Youngstock*ᵇ | 408.1 | 412.9 | 442.4 | 446.3 | 381.0 | 397.7 | 389.6 | 394.5 | 372.4 | 400.4 | 368.7 |
| *Beef Suckling to Youngstock non-rearing*ᵇ | 68.9 | 71.7 | 67.4 | 71.9 | 67.7 | 61.7 | 59.2 | 54.2 | 54.2 | 54.6 | 54.0 |
| **Dairyᵃ** | 876.4 | 1,007.6 | 905.8 | 975.5 | 978.2 | 1,054.9 | 1,041.7 | 1,051.3 | 1,084.5 | 1,111.6 | 1,158.3 |
| *Standard Dairy*ᵇ | 525.6 | 590.8 | 463.7 | 524.8 | 561.3 | 602.4 | 598.5 | 601.3 | 625.4 | 651.0 | 696.1 |
| *Non-rearing Dairy - Contract Rearing*ᵇ | 49.4 | 70.0 | 65.2 | 78.6 | 93.4 | 99.4 | 101.1 | 106.7 | 127.4 | 137.1 | 146.2 |
| *Non-rearing Dairy - No Contract Rearing*ᵇ | 15.9 | 17.4 | 14.5 | 16.0 | 23.0 | 28.2 | 30.7 | 36.8 | 41.2 | 46.3 | 48.1 |
| *Dairy Rearing Male Calves*ᵇ | 285.5 | 329.4 | 362.5 | 356.2 | 300.6 | 325.0 | 311.4 | 306.5 | 290.6 | 277.2 | 267.9 |
| **Fatteningᵃ** | 351.5 | 341.4 | 335.2 | 382.9 | 378.3 | 395.7 | 427.7 | 432.3 | 442.0 | 429.7 | 440.8 |
| **Mixed Productionᵃ** | 274.7 | 293.0 | 256.6 | 277.7 | 289.3 | 313.4 | 321.9 | 350.7 | 334.6 | 357.1 | 340.2 |
| **Store/Rearingᵃ** | 128.1 | 156.8 | 148.8 | 161.5 | 174.7 | 179.6 | 187.4 | 184.2 | 191.5 | 198.6 | 241.4 |
| *Rearing Dairy Females*ᵇ | 3.8 | 5.8 | 5.5 | 7.1 | 8.3 | 8.5 | 9.1 | 9.3 | 12.2 | 13.4 | 14.0 |
| *Store - Beef Females*ᵇ | 26.0 | 30.1 | 25.0 | 29.1 | 31.7 | 31.2 | 32.8 | 34.6 | 36.2 | 35.6 | 41.3 |
| *Store - Beef Males*ᵇ | 43.7 | 49.3 | 46.8 | 48.7 | 51.0 | 49.0 | 50.4 | 50.5 | 51.8 | 51.6 | 61.7 |
| *Store - Beef Mixed*ᵇ | 32.9 | 35.2 | 32.8 | 37.7 | 42.1 | 45.2 | 50.1 | 51.8 | 52.5 | 57.4 | 71.5 |
| *Store - Dairy Males*ᵇ | 21.7 | 36.4 | 38.8 | 38.8 | 41.5 | 45.6 | 44.9 | 38.1 | 38.8 | 40.5 | 53.0 |
| **Trader (Dealer)ᵃ** | 20.3 | 20.7 | 22.4 | 20.2 | 19.5 | 20.6 | 18.9 | 20.2 | 20.3 | 19.0 | 22.1 |
| **Unclassifiedᵃ** | 27.4 | 26.7 | 20.8 | 18.2 | 27.1 | 24.3 | 22.9 | 20.2 | 19.0 | 21.8 | 21.1 |
| **Total** | **2,924.8** | **3,196.7** | **2,892.8** | **3,060.8** | **3,042.3** | **3,206.3** | **3,219.9** | **3,249.9** | **3,251.6** | **3,284.1** | **3,317.1** |

Supplementary Table S10. Sector stock value (€ Million) by a) main herd type and b) herd subtype from 2011 to 2021, adjusted for inflation (Base December 2011=100) (23).

| **Herd Classes** | **2011** | **2012** | **2013** | **2014** | **2015** | **2016** | **2017** | **2018** | **2019** | **2020** | **2021** |
| --- | --- | --- | --- | --- | --- | --- | --- | --- | --- | --- | --- |
| **Beefᵃ** | 2,590.7 | 3,185.0 | 2,696.3 | 2,578.8 | 2,793.7 | 2,558.8 | 2,603.4 | 2,579.7 | 2,452.2 | 2,513.1 | 2,795.7 |
| *Beef Pedigree Herds*ᵇ | 77.6 | 91.2 | 76.1 | 78.1 | 86.0 | 78.4 | 80.7 | 83.8 | 86.2 | 89.8 | 104.2 |
| *Beef Suckling to Beef*ᵇ | 517.3 | 603.2 | 543.2 | 540.5 | 553.6 | 540.8 | 538.0 | 562.6 | 533.6 | 492.5 | 478.6 |
| *Beef Suckling to Weanlings*ᵇ | 998.1 | 1,336.3 | 924.9 | 863.7 | 1,080.6 | 968.0 | 1,005.5 | 957.0 | 926.7 | 932.6 | 1,130.1 |
| *Beef Suckling to Youngstock*ᵇ | 854.0 | 982.9 | 998.3 | 944.7 | 912.0 | 840.7 | 850.1 | 858.9 | 791.3 | 880.3 | 946.7 |
| *Beef Suckling to Youngstock non-rearing*ᵇ | 143.6 | 171.4 | 153.7 | 151.8 | 161.5 | 130.9 | 129.1 | 117.4 | 114.4 | 117.8 | 136.1 |
| **Dairyᵃ** | 1,931.9 | 2,514.0 | 2,130.6 | 2,188.2 | 2,481.1 | 2,354.6 | 2,412.4 | 2,422.7 | 2,439.8 | 2,629.0 | 3,164.6 |
| *Standard Dairy Herd*ᵇ | 1,170.7 | 1,480.8 | 1,091.2 | 1,187.8 | 1,435.2 | 1,353.9 | 1,396.3 | 1,396.5 | 1,418.4 | 1,559.2 | 1,922.4 |
| *Non Rearing Dairy - Contract Rearing*ᵇ | 109.5 | 175.1 | 153.4 | 176.5 | 236.8 | 222.0 | 234.2 | 245.7 | 286.5 | 325.1 | 398.8 |
| *Non Rearing Dairy - No Contract Rearing*ᵇ | 32.2 | 38.8 | 30.6 | 33.2 | 54.0 | 58.9 | 66.2 | 79.5 | 87.0 | 103.4 | 122.0 |
| *Dairy Herd Rearing Male Calves*ᵇ | 619.4 | 819.2 | 855.5 | 790.6 | 755.1 | 719.8 | 715.7 | 701.0 | 647.8 | 641.2 | 721.3 |
| **Fatteningᵃ** | 818.2 | 902.8 | 864.8 | 903.4 | 982.5 | 937.9 | 1,037.2 | 1,043.8 | 1,039.6 | 990.7 | 1,157.1 |
| **Mixed Productionᵃ** | 587.1 | 716.7 | 596.8 | 605.3 | 714.9 | 682.7 | 724.0 | 785.6 | 730.3 | 806.0 | 897.0 |
| **Store/Rearingᵃ** | 291.2 | 417.3 | 381.7 | 375.0 | 457.9 | 417.2 | 448.9 | 441.1 | 446.8 | 459.9 | 662.9 |
| *Rearing Dairy Females*ᵇ | 9.3 | 16.2 | 14.2 | 17.5 | 23.6 | 21.0 | 23.9 | 24.2 | 31.4 | 36.9 | 45.4 |
| *Store - Beef Females*ᵇ | 58.9 | 78.0 | 59.6 | 66.5 | 83.1 | 69.9 | 78.4 | 82.0 | 83.9 | 88.7 | 121.9 |
| *Store - Beef Males*ᵇ | 104.8 | 138.7 | 130.5 | 120.6 | 139.0 | 123.5 | 126.8 | 126.7 | 125.4 | 116.5 | 160.8 |
| *Store - Beef Mixed*ᵇ | 73.4 | 92.6 | 82.3 | 86.3 | 110.0 | 103.3 | 117.8 | 121.2 | 119.6 | 130.6 | 195.6 |
| *Store - Dairy Males*ᵇ | 44.8 | 91.9 | 95.0 | 84.1 | 102.3 | 99.5 | 102.1 | 87.0 | 86.5 | 87.2 | 139.2 |
| **Trader (Dealer)ᵃ** | 43.9 | 50.5 | 52.9 | 44.5 | 48.2 | 45.3 | 42.8 | 45.1 | 44.7 | 42.0 | 57.5 |
| **Unclassifiedᵃ** | 60.7 | 67.8 | 51.3 | 41.1 | 69.2 | 55.4 | 54.0 | 47.3 | 43.3 | 50.4 | 57.7 |
| **Total** | **6,323.7** | **7,854.2** | **6,774.4** | **6,736.3** | **7,547.6** | **7,051.9** | **7,322.7** | **7,365.2** | **7,196.7** | **7,491.0** | **8,792.3** |

Supplementary Table S11. Average herd biomass ('000 kg) by a) main herd type and b) herd subtype from 2011 to 2021.

| **Herd Classes** | **2011** | **2012** | **2013** | **2014** | **2015** | **2016** | **2017** | **2018** | **2019** | **2020** | **2021** |
| --- | --- | --- | --- | --- | --- | --- | --- | --- | --- | --- | --- |
| **Beefᵃ** | 20.5 | 22.5 | 20.1 | 21.0 | 20.7 | 21.8 | 21.9 | 22.1 | 22.2 | 22.4 | 22.1 |
| *Beef Pedigree*ᵇ | 15.6 | 17.4 | 15.9 | 17.2 | 16.8 | 17.6 | 17.8 | 18.1 | 18.7 | 19.0 | 18.8 |
| *Beef Suckling to Beef*ᵇ | 33.0 | 36.0 | 32.4 | 35.1 | 35.3 | 37.9 | 38.8 | 39.2 | 39.3 | 36.8 | 37.0 |
| *Beef Suckling to Weanlings*ᵇ | 15.5 | 17.5 | 14.7 | 14.9 | 15.2 | 16.1 | 16.2 | 16.0 | 16.3 | 16.5 | 16.7 |
| *Beef Suckling to Youngstock*ᵇ | 27.3 | 29.9 | 25.9 | 27.2 | 27.5 | 28.1 | 28.4 | 28.6 | 28.3 | 29.2 | 29.2 |
| *Beef Suckling to Youngstock non-rearing*ᵇ | 16.4 | 18.0 | 15.8 | 16.6 | 17.2 | 16.9 | 16.7 | 16.3 | 16.6 | 16.5 | 17.3 |
| **Dairyᵃ** | 66.2 | 73.5 | 66.1 | 71.4 | 72.8 | 78.7 | 80.5 | 84.6 | 87.6 | 91.1 | 92.9 |
| *Standard Dairy*ᵇ | 63.1 | 69.5 | 61.2 | 67.2 | 68.9 | 75.6 | 77.0 | 82.4 | 84.2 | 89.1 | 90.6 |
| *Non-rearing Dairy - Contract Rearing*ᵇ | 90.7 | 107.0 | 90.5 | 94.3 | 102.2 | 106.6 | 108.9 | 114.9 | 120.7 | 126.8 | 133.0 |
| *Non-rearing Dairy - No Contract Rearing*ᵇ | 29.2 | 32.0 | 29.6 | 29.7 | 34.6 | 37.7 | 39.5 | 42.4 | 45.2 | 48.9 | 49.7 |
| *Dairy Rearing Male Calves*ᵇ | 74.5 | 82.2 | 73.5 | 79.2 | 80.9 | 86.4 | 89.9 | 92.0 | 97.2 | 96.5 | 98.4 |
| **Fatteningᵃ** | 22.6 | 23.6 | 22.4 | 24.0 | 24.9 | 25.0 | 26.2 | 26.4 | 26.6 | 25.9 | 27.0 |
| **Mixed Productionᵃ** | 52.7 | 56.1 | 49.7 | 54.5 | 54.8 | 58.7 | 60.5 | 64.7 | 65.1 | 67.4 | 67.9 |
| **Store/Rearingᵃ** | 10.8 | 11.2 | 10.6 | 11.0 | 11.0 | 11.4 | 11.7 | 11.7 | 11.7 | 12.1 | 13.0 |
| *Rearing Dairy Females*ᵇ | 22.2 | 23.4 | 19.5 | 20.7 | 20.7 | 21.5 | 21.9 | 23.4 | 24.7 | 24.9 | 25.7 |
| *Store - Beef Females*ᵇ | 7.9 | 8.4 | 7.3 | 7.7 | 7.7 | 7.9 | 8.0 | 8.2 | 7.9 | 8.1 | 8.6 |
| *Store - Beef Males*ᵇ | 9.7 | 9.6 | 9.2 | 9.4 | 9.3 | 9.2 | 9.3 | 9.1 | 9.0 | 9.4 | 9.9 |
| *Store - Beef Mixed*ᵇ | 13.9 | 14.8 | 12.9 | 13.6 | 13.6 | 14.3 | 14.8 | 15.3 | 15.4 | 15.5 | 16.5 |
| *Store - Dairy Males*ᵇ | 14.4 | 13.7 | 14.0 | 14.9 | 15.1 | 15.3 | 16.5 | 16.7 | 17.6 | 17.7 | 19.1 |
| **Trader (Dealer)ᵃ** | 22.4 | 21.3 | 22.6 | 21.9 | 20.7 | 22.5 | 22.8 | 25.6 | 26.0 | 23.5 | 29.0 |
| **Unclassifiedᵃ** | 4.8 | 5.3 | 4.8 | 5.0 | 5.8 | 5.9 | 6.1 | 5.2 | 5.4 | 5.5 | 5.7 |

Supplementary Table S12. Average herd stock value (€ '000) by a) main herd type and b) herd subtype from 2011 to 2021, adjusted for inflation (Base December 2011=100) (23).

| **Herd Classes** | **2011** | **2012** | **2013** | **2014** | **2015** | **2016** | **2017** | **2018** | **2019** | **2020** | **2021** |
| --- | --- | --- | --- | --- | --- | --- | --- | --- | --- | --- | --- |
| **Beefᵃ** | 42.7 | 53.1 | 45.1 | 44.3 | 49.2 | 45.8 | 47.5 | 47.9 | 46.9 | 49.0 | 56.4 |
| *Beef Pedigree Herds*ᵇ | 31.7 | 40.3 | 34.7 | 35.4 | 39.3 | 36.3 | 37.8 | 38.3 | 38.7 | 40.9 | 47.3 |
| *Beef Suckling to Beef*ᵇ | 71.1 | 87.8 | 75.6 | 76.7 | 86.7 | 82.8 | 87.4 | 87.9 | 86.0 | 82.3 | 95.6 |
| *Beef Suckling to Weanlings*ᵇ | 31.4 | 40.4 | 31.8 | 30.6 | 35.5 | 32.9 | 34.4 | 34.0 | 33.8 | 35.8 | 42.5 |
| *Beef Suckling to Youngstock*ᵇ | 57.0 | 71.1 | 58.5 | 57.6 | 66.0 | 59.3 | 61.9 | 62.2 | 60.2 | 64.1 | 74.9 |
| *Beef Suckling to Youngstock non-rearing*ᵇ | 34.3 | 43.0 | 36.0 | 35.2 | 41.0 | 35.8 | 36.5 | 35.2 | 35.1 | 35.7 | 43.5 |
| **Dairyᵃ** | 145.9 | 183.5 | 155.4 | 160.1 | 184.7 | 175.6 | 186.4 | 195.0 | 197.1 | 215.4 | 253.8 |
| *Standard Dairy Herd*ᵇ | 140.6 | 174.2 | 144.1 | 152.2 | 176.2 | 169.9 | 179.5 | 191.4 | 190.9 | 213.4 | 250.1 |
| *Non Rearing Dairy - Contract Rearing*ᵇ | 201.0 | 267.7 | 213.1 | 211.9 | 259.1 | 238.2 | 252.2 | 264.5 | 271.4 | 300.8 | 362.7 |
| *Non Rearing Dairy - No Contract Rearing*ᵇ | 59.4 | 71.4 | 62.6 | 61.7 | 81.2 | 78.9 | 85.3 | 91.4 | 95.6 | 109.1 | 126.2 |
| *Dairy Herd Rearing Male Calves*ᵇ | 161.6 | 204.3 | 173.5 | 175.8 | 203.1 | 191.3 | 206.7 | 210.3 | 216.8 | 223.2 | 265.1 |
| **Fatteningᵃ** | 52.5 | 62.4 | 57.9 | 56.6 | 64.8 | 59.2 | 63.6 | 63.6 | 62.5 | 59.7 | 71.0 |
| **Mixed Productionᵃ** | 112.6 | 137.2 | 115.6 | 118.9 | 135.5 | 127.9 | 136.1 | 144.9 | 142.1 | 152.0 | 179.0 |
| **Store/Rearingᵃ** | 24.7 | 29.8 | 27.1 | 25.6 | 28.9 | 26.4 | 28.1 | 28.0 | 27.3 | 28.1 | 35.6 |
| *Rearing Dairy Females*ᵇ | 54.6 | 65.6 | 50.9 | 51.0 | 58.8 | 53.0 | 57.1 | 61.0 | 63.5 | 68.5 | 83.9 |
| *Store - Beef Females*ᵇ | 17.9 | 21.8 | 17.4 | 17.6 | 20.2 | 17.7 | 19.1 | 19.5 | 18.4 | 20.3 | 25.4 |
| *Store - Beef Males*ᵇ | 23.3 | 26.9 | 25.7 | 23.3 | 25.2 | 23.2 | 23.4 | 22.9 | 21.7 | 21.2 | 25.8 |
| *Store - Beef Mixed*ᵇ | 31.0 | 38.8 | 32.5 | 31.2 | 35.4 | 32.5 | 34.8 | 35.7 | 35.0 | 35.2 | 45.1 |
| *Store - Dairy Males*ᵇ | 29.8 | 34.5 | 34.2 | 32.2 | 37.2 | 33.3 | 37.6 | 38.2 | 39.2 | 38.1 | 50.1 |
| **Trader (Dealer)ᵃ** | 48.2 | 52.0 | 53.3 | 48.0 | 51.2 | 49.3 | 51.5 | 57.2 | 57.0 | 51.9 | 75.4 |
| **Unclassifiedᵃ** | 10.8 | 13.4 | 11.8 | 11.3 | 14.8 | 13.4 | 14.5 | 12.2 | 12.3 | 12.7 | 15.4 |
